# Exploring the power of data mining for uncovering traditional medicinal plant knowledge: A case study in Shahrbabak, Iran

**DOI:** 10.1371/journal.pone.0303229

**Published:** 2024-06-10

**Authors:** Hossein Bibak, Farzad Heydari, Mohammad Sadat-Hosseini

**Affiliations:** 1 Faculty of Science, Department of Biology, University of Jiroft, Jiroft, Iran; 2 Faculty of Mathematics and Computer, Department of Computer Science, Shahid Bahonar University of Kerman, Kerman, Iran; 3 Faculty of Agriculture, Department of Horticultural Science, University of Jiroft, Jiroft, Iran; University of Poonch Rawalakot, PAKISTAN

## Abstract

The present study recorded indigenous knowledge of medicinal plants in Shahrbabak, Iran. We described a method using data mining algorithms to predict medicinal plants’ mode of application. Twenty-oneindividuals aged 28 to 81 were interviewed. Firstly, data were collected and analyzed based on quantitative indices such as the informant consensus factor (ICF), the cultural importance index (CI), and the relative frequency of citation (RFC). Secondly, the data was classified by support vector machines, J48 decision trees, neural networks, and logistic regression. So, 141 medicinal plants from 43 botanical families were documented. Lamiaceae, with 18 species, was the dominant family among plants, and plant leaves were most frequently used for medicinal purposes. The decoction was the most commonly used preparation method (56%), and therophytes were the most dominant (48.93%) among plants. Regarding the RFC index, the most important species are *Adiantum capillus-veneris* L. and *Plantago ovata* Forssk., *while Artemisia auseri* Boiss. ranked first based on the CI index. The ICF index demonstrated that metabolic disorders are the most common problems among plants in the Shahrbabak region. Finally, the J48 decision tree algorithm consistently outperforms other methods, achieving 95% accuracy in 10-fold cross-validation and 70–30 data split scenarios. The developed model detects with maximum accuracy how to consume medicinal plants.

## Introduction

The utilization of plants for traditional medicine and health purposes has been around since ancient times and is becoming increasingly popular in many parts of the world [1,2]. Medicinal plants are rich in effective substances that treat various diseases [3]. For novel drug development, the first and most critical stage is the collection and analysis of information on medicinal plants used by various indigenous cultures [4]. Ethnopharmacological studies are necessary to obtain the past and present state of cultural habits about plants around the world. It is also essential to record indigenous people’s knowledge of medicinal plants [1,5,6]. Furthermore, it supports the preservation of traditional knowledge for future generations and other communities [7,8]. Throughout Iran, ethnopharmacological studies have been conducted on plants for many years [9–16]. With 900,000 ha of natural resources, the Shahrbabak has many medicinal plants. However, the existing literature indicates a significant gap in our understanding of how local populations utilize these species for medicinal purposes and disease treatment.

Data mining emerged in the mid-1990s as a method for uncovering hidden knowledge. Data mining can identify complexity, discover potential causal relationships, and find hidden relationships, and correlations between variables [17].

- Data mining is the science of extracting patterns, information, and analysis from raw datasets produced by an organization, a society, or any other set. Data mining transforms useless information into useful information by obtaining valuable results. At a more detailed level, data mining is a step in the Knowledge Discovery in Databases (KDD) process. Generally, four stages or main steps can be considered for data mining: determining goals, collecting and preparing data, extracting patterns, and evaluating the results [18]. Data mining algorithms are divided into four categories based on performance: **Classification (supervised learning):** In this type of learning, a set of samples with their labels is provided to the model, and the model must establish a relationship between the examples and their labels. This algorithm can learn from the labeling model and use data mining algorithms to label and separate new samples. Classification algorithms include decision trees, support vector machines, neural networks, and logistic regression.
- **Clustering (unsupervised learning):** In this case, the algorithm divides the data into groups based on their similarities. Unsupervised learning uses unlabeled samples. In these algorithms, a cost function and a distance measurement are defined. The algorithms should reduce the cost function value according to distance measurement.
- **Semi-supervised learning** involves labeled and unlabeled data. Semi-supervised learning methods are somewhere between unsupervised and supervised learning methods.
- **Reinforcement learning**, the algorithm continuously discovers and learns by exchanging information and operations with the surrounding environment. When a machine receives a reward, it can learn how to improve itself to receive more rewards in the future. This is done by performing specific actions [19].

We used different data mining algorithms for prediction. The most crucial classification algorithms used in this article are described below.

- **Decision tree** is a supervised learning (classification) method. A decision tree has a structure where an internal node represents an attribute, a branch means a decision rule, and each leaf node indicates an outcome. The highest node in the decision tree is known as the root node, which is the highest level of the tree. A decision tree is suitable for establishing non-linear relationships between features and classes. The decision tree is flexible because it can easily model non-linear or unconventional relationships. It can interpret the interaction between predictors. This method can also be well interpreted due to its binary structure [19,20].

**Support Vector Machine** is a supervised learning algorithm that controls and solves classification problems. This algorithm is applied to different classification fields. SVM is designed to achieve the goal of class members having the least distance from each other and the maximum length from other classes. This technique is a supervised learning model used for linear and non-linear classification. The basis of the work of SVM classifiers is the linear classification of the data. In the linear division of the data, an attempt is made to select a line with a higher margin of confidence [19,20].

**Logistic regression** is a classification algorithm that assigns observed samples to a distinct set of classes. Unlike linear regression, which produces continuous numerical values, it uses the logistic sigmoid function to transform its output to return a probability value that can be mapped into two or more distinct classes. Logistic regression works well when the data relationship is almost linear but poorly if non-linear relationships exist between the variables [19,20].

**Artificial Neural Networks (ANN)** is an information processing paradigm inspired by biological neural systems such as the brain that process information. ANN consists of several layers of simple processing elements called neurons. The neuron performs the two functions of collecting inputs and producing an output. Using ANN provides an overview of the theory, learning rules, and applications of the most important neural network models, definitions, and computational styles [21].

Data quality is vital in data analysis because incorrect data leads to wrong results. Fast detection of data quality issues reduces the effort and time needed to find and analyze them. Therefore, it is necessary to use data mining methods to find defects and fix wrong data [22].

Data mining starts with raw data and continues until new knowledge is formed. Data cleansing refers to identifying, removing, and correcting wrong data from tables, records, or databases. It also includes identifying incomplete and incorrect data parts and correcting and replacing them. Data Integration is collecting data from multi-source systems to create single sets of information for operational and analytical applications. During the Data Selection section, the dataset should be selected and retrieved. Sometimes, to increase the accuracy of the analysis, we have to change the raw data available for analysis. One of these changes is the Data Transformation process [23]. In addition, we need to identify the right features. Choosing the most critical features improves the efficiency of data mining algorithms and data understanding, reduces algorithm execution time, reduces data storage volume, and simplifies the model. Feature selection methods are divided into Filters, Wrappers, and Embedded [24].

Very few studies have been performed on data mining methods to increase the discovery of hidden knowledge of ethnopharmacology. It has been found that data mining in ethnopharmacology has two crucial advantages. First, it utilizes qualitative and quantitative data (such as observations and sensor information) to study practically inaccessible phenomena through each data type alone. Second, it provides a means of interpreting that data, which produces novel insights by exposing the biases inherent in each data type alone [25]. Axiotis et al. performed a study using an intelligent search system to support ethnopharmacological research through a combination of active learning and reinforcement learning. They reported that Machine learning-powered research improved the effectiveness and efficiency of the domain expert by 3.1 and 5.14 times, respectively. This was done by fetching 420 relevant ethnopharmacological documents in only seven hours versus an estimated 36 hours of human effort [26]. The current study documented ethnomedicinal knowledge of medicinal plants in the Shahrbabak region in the southeastern part of Iran, within Kerman Province. This work analyzed medicinal plants used for treating various diseases. Also, for the first time, we recommend modeling and comparing data mining algorithms to predict medicinal plant modes of application. Our study combines qualitative ethnobotanical fieldwork with advanced data mining approaches to create a systematic framework for collecting and analyzing traditional medicinal plant data. Also, the current study demonstrates the potential of data mining as a tool for unlocking valuable insights from traditional knowledge systems.

## Material and methods

### Study area

The city of Shahrbabak is situated (30° 11ʹ63ʺ N 55° 11ʹ86ʺ E) North-West of Kerman Province with an area of 13500 square kilometers and an altitude of 1845 m above sea level. According to the 2006 Iranian census, this area had 43,916 residents. Shahrbabak is an ancient Iranian city. Meymand, one of Iran’s four ancient villages, is 36 kilometers from Shahrbabak. This town is near Sarcheshmeh and Miedook, Iran’s largest copper mines. Historians say this town was built by the Sassanid king Ardeshir Babakan 1800 years ago. Shahrbabak has a semiarid climate with hot and dry summers and cold and dry winters. This region’s annual temperature, average rainfall, and humidity ranges are 16.2°C, 162 mm, and 34%, respectively.

### Collecting data and identifying plants

Data were collected from different parts of the Shahrbabak district, North-West of Kerman Province. The interviewees were identified as indigenous practitioners, sellers, shepherds and medicinal herb vendors who assisted with identifying plants they regarded as medicinal (the questionnaire is accessible through an online Supplementary file). Twenty-oneindividuals (11 females and 10 males) aged 28 to 81 were interviewed. Plants were collected from Robat, Meymand, Khatoun Abad, Estabragh, Mehrabad, Dehej-Jowzam, Abdar, Barfe, and Khabr regions, all parts of the Shahrbabak district. Information on vernacular names, herbal part(s) as pharmacological agents, medicinal uses, methods of treatment and preparation was recorded, shown in Table 1. The plants were dried, labeled, and preserved in the Herbarium of the Biology Department at the University of Jiroft for identification and future work. Medicinal plants were identified using Iranica flora [27], Palestine flora [28], Iraq flora [29], Turkey flora [30], and Iran flora (in color) [31]. Plant life cycles were classified according to Raunkiaer’s system [32].

**Table 1:** Indigenous medicinal knowledge of plants from the study area.

(Code) Family	Scientific name	Vernacular name (Persian)	Plant part used	Medicinal uses	Preparation	Mode of application	Life-form	Voucher no.
(1) *Amaranthaceae*	*Anabasis aphylla* L.	Andarouk	Aerial parts	Constipation	Infusion	Oral	Th	125
(2) *Amaranthaceae*	*Dysphania botrys* (L.) Mosyakin & Clemants	Dermeneh	Leaf and flower	Skin rash	Liniment	Topical	C	137
(3) *Amaryllidaceae*	*Narcissus tazetta* L.	Narges	Corm	Remove inflammation of the skin, nerve tonic	Decoction	Oral	Th	467
(4) *Anacardiaceae*	*Pistacia khinjuk* Stocks.	Kasour	Oil of fruit	Anti-bacterial	Decoction, liniment	Oral, Topical	P	641
(5) *Apiaceae*	*Ridolfia segetum (*Guss.) Moris	Meytokhm	Fruit and leaf	Stomach ulcers, Appetizing	Decoction	Oral	P	234
(6) *Apiaceae*	*Elwendia persica *(Boiss.) Pimenov & Kljuykov	Ziresiah	Seed and leaf	Digestive, menstruation	Decoction	Oral	Th	227
(7) *Apiaceae*	*Carum carvi* L.	Ziresiah	Fruit	Digestive system	Infusion	Oral	Th	144
(8) *Apiaceae*	*Cuminum cyminum* L.	Ziresabz	Whole plant	Carminative	Decoction	Oral	Th	225
(9) *Apiaceae*	*Coriandrum sativum* L.	Geshniz	Leaf, fruit	Anti—tumult in stomach	Decoction	Oral	Th	238
(10) *Apiaceae*	*Daucus carota* L	Havij	Root, fruit	Eye tonic, blood problem	Decoction	Oral	H	148
(11) *Apiaceae*	*Ferula ammoniacum (D*.*Don) Spalik*, *M*.*Panahi*, *Piwczyński & Puchałka*	Oshtork	Resin	Regulatory of Menstruation, sudorific	Decoction	Oral	H	258
(12) *Apiaceae*	*Ferula aucheri (Boiss*.*) Piwczyński*, *Spalik*, *M*.*Panahi & Puchałka*	Bekhar	Resin, stem	Epilepsy, anti-cancer	Decoction (with yogurt)	Oral	H	249
(13) *Apiaceae*	*Ducrosia anethifolia* (DC.)Boiss.	Reshgak	Fruit, seed	Carminative in children	Decoction	Oral	Th	291
(14) *Apiaceae*	*Ducrosia assadii* Alava.	Reshgak	Oil of Fruit and leaf	Wound healing, burn	Liniment	Topical	Th	155
(15) *Apiaceae*	*Ferula assa-foetida* L.	Anghouzeh	Resin	Menstruation	Decoction	Oral	G	157
(16) *Apiaceae*	*Ferula gummosa* Boiss.	Anghouzah	Resin	Cough, laxative	Decoction	Oral	G	236
(17) *Apiaceae*	*Ferula ovina (Boiss*.*) Boiss*.	Anghouzake shirin	Resin	Intestinal infections	Powder	Oral	G	159
(18) *Apiaceae*	*Foeniculum vulgare* Mill.	Badioun	Fruit	Carminative, cough, asthma, food digestion	Decoction	Oral	Th	244
(19) *Apiaceae*	*Heracleum persicum* Desf. ex Fisch., C.A.Mey. & Avé-Lall	Golpar	Fruit, leaf, flower	Nerve tonic	Moisturized in water	Oral	Th	269
(20) *Apiaceae*	*Petroselinum crispum* (Mill.) Fuss.	Jafari	Aerial parts	Iron supply	Freshly cooked	Oral	Th	229
(21) *Apocynaceae e*	*Nerium oleander* L	Gish	Leaf	Abdominal pains	Moisturized in water	Oral	C	861
(22) *Asparagaceae*	*Muscari comosum *(L.) Mill.	Sirmouk	Corm	Pertussis, adult squirt, bronchitis	Decoction	Oral	G	190
(23) *Asteraceae*	*Achillea eriophora* DC.	Boumadaran	Flower, Leaf	Digestive, abdominal pains diarrhea, menstrual pains	Decoction	Oral	Th	921
(24) *Asteraceae*	*Achillea wilhelmsii *K.Koch	Boumadaran	Aerial parts	Digestive, abdominal pains diarrhea, menstrual pains	Decoction	Oral	Th	918
(25) *Asteraceae*	*Artemisia aucheri* Boiss.	Jaz	Oil of aerial parts	Skin and hair disorders	Liniment	Topical	C	934
(26) *Asteraceae*	*Cichorium pumilum* Jacq.	Kasni	Juice of root	Heat of body, Liver problem, jaundice	Decoction	Oral	Th	913
(27) *Asteraceae*	*Cichorium intybus* L.	Kasni	Juice of root	Heat of body, Liver problem, jaundice	Decoction	Oral	G	924
(28) *Asteraceae*	*Cirsium arvense* (L.) Scop.	Kangar	Stem	Fever, appetizing	Decoction	Oral	G	925
(29) *Asteraceae*	*Glebionis coronaria (L*.*)* Cass. ex Spach.	Davoodi	Aerial parts	Eye problem, blood purifier	Decoction	Topical	Th	165
(30) *Asteraceae*	*Gundelia tournefortii* L.	Kangar	Stem	Gastric discomfort, constipation	Decoction	Oral	G	974
(31) *Asteraceae*	*Hertia intermedia* (Boiss.) Kuntze	Golghich	Leaf, latex	Adult squirt, parasite repellent	Decoction	Topical	C	973
(32) *Asteraceae*	*Pentanema britannica *(L.) D.Gut.Larr., Santos-Vicente, Anderb., E.Rico & M.M.Mart.Ort.	Mosafa	Leaf	Syrup	Liniment	Oral	Th	167
(33) *Asteraceae*	*Lactuca serriola* L.	Kahouzomokhtou	Aerial parts, latex	Aperients	Decoction	Oral	Th	916
(34) *Asteraceae*	*Launaea acanthodes* (Boiss.) Kuntze	Charkhe	Aerial parts, latex	Sedative	Decoction	Oral	G	169
(35) *Asteraceae*	*Tanacetum parthenium* (L.) Sch.Bip	Babouneh	Aerial parts	Stomach ulcers, gastritis	Decoction	Oral	Th	960
(36) *Berberidaceae*	*Berberis jamesiana* Forrest & W.W.Sm.	Zarch	Fruit, flower	Heat of body, gallbladder diseases	Decoction	Oral	P	681
(37) *Boraginaceae*	*Anchusa azurea* Mill.	Gavzaban	Flower	Nerve tonic	Decoction	Oral	Th	170
(38) *Boraginaceae*	*Echium amoenum* Fisch. & C.A.Mey.	Golgavzaban	Flower	Nerve tonic	Decoction	Oral	Th	734
(39) *Boraginaceae*	*Nonea persica* Boiss.	Serkouei	Flower, leaf	Body detoxification	Decoction	Oral	Th	177
(40) *Boraginaceae*	*Descurainia sophia* (L.)Webb ex Prantl	Hochou	Root	Skin rash, skin burn	Liniment	Topical	Th	178
(41) *Brassicaceae*	*Brassica rapa* L.	Shalgham	Root	Cough, influenza	Decoction,	Oral	Th	749
(42) *Brassicaceae*	*Capsella bursa-pastoris* (L.) Medik.	Keshishou	Aerial parts	Blood coagulation	Decoction, liniment	Oral, topical	Th	185
(43) *Brassicaceae*	*Descurainia sophia* (L.) Webb. ex Prantl.	Khakshir	Seed	Heat of body, constipation	Mix seeds with water	Oral	Th	762
(44) *Brassicaceae*	*Eruca vesicaria* (L.) Cav.	Mendou	Leaf, stem	Tonic	Decoction, Raw eating	Oral	Th	755
(45) *Brassicaceae*	*Lepidium draba* L.	Mokou	Leaf, seed, stem	Anemia	Decoction	Oral	Th	759
(46) *Brassicaceae*	*Lepidium latifolium* L.	Tartizak	leaf	Tonic	Raw eating	Topical	Th	770
(47)*Brassicaceae*	*Lepidium spinosum *Ard.	Shahi	Leaf	Vitamins and minerals	Raw eating	Topical	Th	776
(48) *Brassicaceae*	*Sisymbrium irio* L.	Khakshir	Seed	Adult squirt, constipation	Mix seeds with water	Oral	Th	774
(49) *Caryophyllaceae*	*Herniaria hirsuta L*	Fetgh	Aerial parts	Burn	Liniment	Topical	Th	130
(50) *Convolvulaceae*	*Convolvulus arvensis* L.	Pichak	whole plant	Asthma	Decoction	Oral	G	871
(51) *Cucurbitaceae*	*Citrullus colocynthis* (L) Schrad.	Hanzal	Seed	Bone and joint pains	Liniment	Topical	G	890
(52) *Cucurbitaceae*	*Cucurbita pepo L*.	Kadou	Seed	Constipation	Decoction	Oral	Th	885
(53) *Cuperssaceae*	*Juniperus excelsa M*.*Bieb*.	Ouros	Gum	Rheumatism, bone and joint pains	Liniment	topical	P	203
(54) *Elaeagnaceae*	*Elaeagnus angustifolia* L.	Shesht	Fruit	Abdominal pains diarrhea	Freshly cooked	Oral	P	544
(55) *Ephedraceae*	*Ephedra distachya* L.	Houm	Fruit, stem	Nerve tonic, anaesthetic	Liniment	Topical	C	182
(56) *Ephedraceae*	*Ephedra intermedia* Schrenk & C.A.Mey.	Khimouk	Fruit, stem	Lipotropic	Decoction	Oral	C	179
(57) *Ephedraceae*	*Ephedra sarcocarpa* Aitch. & Hemsl.	Houmnar	Fruit, stem	Eatable color	-	Oral	C	125
(58) *Euphorbiaceae*	*Euphorbia buhsei* Boiss.	Shirsag	Leaf	Anemia, hemophilia	Decoction	Oral	G	126
(59) *Euphorbiaceae*	*Ricinus communis* L.	Kenton	Seed	Hair tonic	Liniment	Topical	G	499
(60) *Fabaceae*	*Alhagi maurorum* Medik.	Kharshotor	Aerial parts	Urinary stone, slimming	Decoction	Oral	G	675
(61) *Fabaceae*	*Alhagi pseudalhagi* (M. Bieb.) Desv. ex B. Keller & Shap.	Kharshotor	Aerial parts	Urinary disease	Decoction	Oral	G	674
(62) *Fabaceae*	*Astragalus eremophilus* Boiss.	Kaliliomolk	Seed	Prevention of blood coagulation, throat pains	Freshly cooked	Oral	C	681
(63) *Fabaceae*	*Astragalus ovoideus* Širj. & Rech.f.	Margin	Gum	Nerve tonic	Decoction	Oral	C	685
(64) *Fabaceae*	*Cercis siliquastrum L*.	Arghavan	Leaf, stem	Gastric discomfort	Decoction	Oral	P	147
(65) *Fabaceae*	*Cicer arietinum* L.	Nokhod	Seed	Intestinal infections	Decoction	Oral	Th	659
(66) *Fabaceae*	*Genista tinctoria* L.	Rangine	Flower Leaf, seed	Constipation	Decoction	Oral	Th	150
(67) *Fabaceae*	*Glycerrhiza glabra* L.	Shirinbayan	Root	Nerve tonic, ulcer	Decoction	Oral	G	650
(68) *Fabaceae*	*Medicago sativa* L.	Yonjeh	Aerial parts	Decrease blood sugar level	Decoction	Oral	G	132
(69) *Fabaceae*	*Melilotus officinalis* (L.) Pall.	Yonjeh baghi	Aerial parts	Appetizing	Decoction	Oral	Th	697
(70) *Fabaceae*	*Ononis spinosa* L.	Kharzomokhtou	Flower, leaf, root	Urinary infection	powder, decoction	Oral	C	134
(71) *Fabaceae*	*Pisum sativum* L.	Nokhod	Seed	Supply of vitamin	Decoction	Oral	Th	136
(72) *Fabaceae*	*Sophora alopecuroides* L.	Talkheh	Seed	Sedative, constipation	Decoction	Oral	G	140
(73) *Fabaceae*	*Trifolium pratense* L.	Sabouei	Seed	Blood purifier, burn	Decoction, Liniment	Topical,oral	Th	693
(74) *Fabaceae*	*Trigonella foenum-graecum* L.	Shanbalile	Leaf, stem	Diabetes	Freshly cooked	Topical	Th	694
(75) *Fabaceae*	*Vicia faba L*.	Baghala	Seed	Constipation, blood fat	Decoction	Oral	Th	689
(76) *Fabaceae*	*Vicia sativa L*.	Mash	Seed	Abdominal pains diarrhea	Decoction	Oral	Th	143
(77) *Gentianaceae*	*Centaurium pulchellum *subsp.* grandiflorum *(Batt.) Maire	Ghantorion	Aerial parts	Anti pyretic, carminative, tonic	Decoction	Oral	Th	195
(78) *Geraniaceae*	*Erodium cicutarium (L*.*) L’Hér*.	Souzan kalaghou	Aerial parts	Adult squirt, intestinal infections	Decoction	Oral	Th	196
(79) *Geraniaceae*	*Geranium rotundifolium L*.	Souzani	Aerial parts	Adult squirt, intestinal infections	Decoction	Oral	G	197
(80) *Iridaceae*	*Iris × germanica* L.	Zanbagh	Rhizome	Rheumatism	Decoction	Oral	G	314
(81) *Juglandaceae*	*Juglans regia L*.	Gerdou	Leaf, fruit, skin	Blood cholesterol, bone and joint pains	Liniment, Decoction	Topical, oral	P	1001
(82) *Lamiaceae*	Ajuga *chamaecistus* Ging. ex Benth.	Samsak	Aerial parts	Urinary infection, anti fungal	Freshly cooked	Oral	C	1010
(83) *Lamiaceae*	*Clinopodium graveolens* (M.Bieb.) Kuntze.	Malangou	Fruit	Stomach bleeding, toxication	Decoction	Oral	Th	1015
(84) *Lamiaceae*	*Dracocephalum polychaetum* Bornm	zarow	Aerial parts	Toothache, Headache, stomachache	Decoction	Oral	Th	231
(85) *Lamiaceae*	*Dracocephalum royleanum *Benth.	Malangou	Fruit	Dysentery	Decoction	Oral	Th	1016
(86)*Lamiaceae*	*Mentha longifolia* (L.) L.	Poudene	Aerial parts	Digestive problem	Decoction	Oral	G	827
(87) *Lamiaceae*	*Nepeta cataria* L.	Nana	Aerial parts	Abdominal pains	Decoction	Oral	Th	1017
(88) *Lamiaceae*	*Nepeta ispahanica* Boiss.	Golzoufa	Aerial parts	Anti–tumult, anti-fungal	Decoction	Oral	Th	1018
(89) *Lamiaceae*	*Nepeta supina* Steven.	Makhlase	Aerial parts	Nausea	Decoction	Oral	H	1019
(90) *Lamiaceae*	*Nepeta glomerulosa* Boiss.	Badrang	Whole plant	Stomach bleeding	Decoction	Oral	H	1020
(91) *Lamiaceae*	*Ocimum basilicum* L.	Reyhan	Aerial parts	Carminative, food digestion	Freshly cooked	Oral	G	835
(92) *Lamiaceae*	*Rydingia persica* (Burm. f.) Scheen & V. A. Albert.	Golder	Leaf, flower	Liver cysts	Infusion	Oral	C	822
(93) *Lamiaceae*	*Salvia macrosiphon* Boiss.	Moureshk	Seed	Anti-bacterial	Decoction	Oral	G	812
(94) *Lamiaceae*	*Teucrium polium* **L.**	Kalpoure	Aerial parts	Tonic, epilepsy	Decoction	Oral	G	819
(95) *Lamiaceae*	*Thymus fedtschenkoi* Ronneger	Avishan	Aerial parts	Influenza	Decoction	Oral	G	828
(96) *Lamiaceae*	*Ziziphora clinopodioides* Lam.	Alaleh	Aerial parts	Never tonic, influenza	Infusion	Oral	Th	804
(97) *Lamiaceae*	*Ziziphora tenuior* L.	Kakuti	Aerial parts	Digestive problem	Decoction	Oral	Th	803
(98) *Lamiaceae*	*Linum usitatissimum* L.	Katan	Seed	Slimming	Infusion	Oral	C	1021
(99) *Malvaceae*	*Althaea aucheri* Boiss.	Khatmi	Flower	Remove rash from skin, backache	Liniment	Topical	Th	584
(100) *Malvaceae*	*Malva parviflora *var.* Parviflora*	Khatmi	Flower, seed	Sedative, anti–tumult, disinfectant	Decoction	Oral	Th	586
(101) *Malvaceae*	*Malva neglecta* Wallr.	Khatmisefid	Flower	Stomach bleeding	Infusion	Oral	Th	1022
(102) *Malvaceae*	*Morus alba* L.	Toot	Fruit	Diuretic	Freshly cooked	Oral	P	1025
(103) *Malvaceae*	*Morus nigra* L.	Shahtoot	Fruit	Diuretic infection	Infusion	Oral	P	1027
(104) *Nitrariaceae*	*Peganum harmala* L.	Dashti	Seed	Anti-bacterial	Smoke	-	Th	573
(105) *Oleaceae*	*Jasminum officinale* L.	Yas	Flower	Sedative, anti-virus	Infusion	Oral	P	1030
(106) *Onagraceae*	*Epilobium angustifolium* L.	Bid	Leaf, root	Cardiac distress, febrifuge	Decoction	Oral	Th	1037
(107) *Onagraceae*	*Epilobium hirsutum* L.	Bid	Leaf, root	Never tonic	Decoction	Oral,	Th	1039
(108) *Papaveraceae*	*Fumaria parviflora* Lam.	Shahtere	Leaf, flower	Sedative, blood purifier	Decoction	Oral	Th	549
(109) *Papaveraceae*	*Papaver dubium* L.	Khaskhashah	Flower, capsule	Sedative	Decoction, liniment	Oral, Topical	Th	629
(110) *Plantaginaceae*	*Plantago lanceolata* L.	Tangbad	Seed	Constipation	Decoction	Oral	Th	490
(111) *Plantaginaceae*	*Plantago major* L.	Kowchak	Leaf, seed, root	Cough, throat infection	Infusion	Oral	Th	492
(112) *Plantaginaceae*	*Plantago ovata* Forssk	Tokhmsefid	Seed	Constipation, anti-leg tumult	Infusion	Oral	Th	1040
(113) *Poaceae*	*Cymbopogon schoenanthus* (L.) Spreng	Kabo	Aerial parts	Skin whitening	Decoction	Topical	G	1045
(114) *Poaceae*	*Hordeum vulgare *subsp.* distichon *(L.) Körn.	Jow	Seed	Febrifuge	Powder	Oral	Th	1046
(115) *Poaceae*	*Phragmites australis* (Cav.) Trin. ex Steud.	Ney	Root	Flavor	Freshly cooked	Oral	G	1048
(116) *Polygonaceae*	*Rheum ribes* L.	Rivas	Leaf, stem	Never tonic, cardiac distress, Blood coagulation	Freshly cooked, syrup	Oral	G	432
(117) *Primulaceae*	*Lysimachia maritima* (L.) Galasso, Banfi & Soldano	Shabdari	Aerial parts	Epilepsy, asthma	Decoction	Oral	Th	1050
(118) *Primulaceae*	*Primula capitellata* Boiss.	Pamchal	Leaf, root	Parasite repellent, epilepsy	Infusion	Oral	Th	1055
(119) *Pteridaceae*	*Adiantum capillus-veneris* L.	Parsiavashoun	Leaf	Influenza, throat infection	Decoction	Oral	G	224
(120) *Ranunculaceae*	*Ranunculus arvensis* L.	Alaleh	Flower	Urinary stone, liver cleaner	Infusion	Oral	G	1060
(121) *Rhamnaceae*	*Ziziphus jujuba* Mill.	Anab	Fruit	Pertussis	Infusion	Oral	P	1065
(122) *Rosaceae*	*Prunus amygdalus *Batsch	Badam	Fruit	Hair tonic	Liniment	Topical	P	360
(123) *Rosaceae*	*Cotoneaster persicus* Pojark.	Siahchou	Fruit	Constipation, cough	Decoction	Topical	P	369
(124) *Rosaceae*	*Crataegus ambigua* C.A.Mey. ex A.K.Becker	Kalkouhi	Fruit	Gastric discomfort	Freshly cooked	Oral	P	365
(125) *Rosaceae*	*Prunus scoparia* (Spach) C. K. Schneid.	Ghousak	Fruit, flower, seed	Skin rash, hair tonic, cough	Liniment	Topical	P	376
(126) *Rosaceae*	*Rosa canina* L.	Korik	Fruit, flower, leaf	Never tonic	Infusion	Oral	P	362
(127) *Rosaceae*	*Rosa × damascena Herrm*.	Golmohamadi	flower	Detoxification, diabetes, never tonic	Infusion	Oral	P	1070
(128) *Rosaceae*	*Rubus caesius* L.	Temeshk	Fruit, flower	Heat of body	Infusion	Oral	C	371
(129) *Rosaceae*	*Sanguisorba minor* Scop.	Gheytaran	Aerial parts	Adult squirt, burn	Powder	Oral, topical	Th	379
(130) *Rubiaceae*	*Rubia albicaulis Boiss*.	Ruunask	Fruit	Constipation	Decoction	Oral	Th	1075
(131) *Rubiaceae*	*Rubia tinctorum L*.	Runas	Root	Constipation, fracture	Decoction	Oral	Th	1078
(132) *Salicaceae*	*Salix mucronata *Thunb.	Bidmeshk	Flower	Digestive, menstruation additive, adult squirt	Infusion	Oral	P	699
(133) *Salicaceae*	*Populus alba L*.	Sepidar	Leaf, skin	Sedative, dilution of blood	Decoction	Oral	P	1080
(134) *Solanaceae*	*Datura stramonium* L.	Tature	Leaf, seed	Sexual desire	Decoction	Oral	Th	381
(135) *Solanaceae*	*Hyoscyamus reticulatus* L.	Benji	Aerial parts	Sedative	Infusion	Oral	P	389
(136) *Solanaceae*	*Lycium shawii *Roem. & Schult.	Zil	Fruit	Child squirt	Freshly cooked	Oral	C	386
(137) *Thymelaeaceae*	*Diarthron lessertii* (Wikstr.) Kit Tan.	Golbidi	Leaf, flower	Arthritis, bone and joint pains, Liver problem	Infusion	Oral	Th	1085
(138) *Urticaceae*	*Urtica urens* L.	Soznaku	Leaf	Toothache	Infusion	Oral	G	321
(139) *Xanthorrhoeaceae*	*Eremurus persicus* (Jaub. & Spach) Boiss	Srishou	Root	Liver problem, digestive	Powder	Oral	G	453
(140) *Zygophyllaceae*	*Kallstroemia maxima *(L.) Hook. & Arn.	Kharkhasak	Fruit	Urinary stone, dilution of blood	Infusion	Oral	Th	574
(141) *Zygophyllaceae*	*Zygophyllum fabago* L.	Ghich	Seed	Child squirt, parasite repellent	Powder	Oral	P	1090

H, hemicryptophytes; G, geophytes; Th, therophytes; P, phanerophytes; C, chamaephytes.

### Data analysis

Ethnomedicine information was evaluated using plant medicinal reports. Three variables were used to define this indicator: i, u, and s. Accordingly, the informant ‘**i**’ mentions the use of species ‘**s**’ in a specific category of use ‘**u**’. The number of medicinal plants and the number of informants reporting the use of a species were counted. We also calculated quantitative value indices.

### Informant consensus factor (ICF)

The Informant Consensus Factor (ICF) was applied to determine the homogeneity of information. The claims regarding medicinal uses are termed ‘citations’, which were classified into ailment categories where each plant was deemed adequate. The ICF index was estimated as follows:

ICF=Nur‐Nt/Nur‐1

Here, ‘Nur’ represents the number of citations used in each category whereas ‘Nt’ represents the number of species used for medicinal purposes [33].

The following formula was employed to compute the relative frequency of citation (RFC) index:

RFC=FC/N

This index (RFC) was computed when the frequency of citation (FC) (i.e. the number of interviewees who mentioned a beneficial species) was divided by the total number of participants in the survey (N). The RFC index ranged from 0 (when no informants mentioned it as beneficial) to 1 (when all interviewees revealed it as beneficial).

Using the following equation, the cultural importance index (CI) was estimated:

$$CI=\sum_{u=u1}^{u_{NC}}\sum_{i-i1}^{i_{N}}\frac{{UR}_{ui}}{N}$$

A CI index considers the frequency of use of a species (according to the number of informants) and the number of cases in which it is used.

The correlation between an informant’s age and the number of applications reported by each informant mentioned for a given medicinal plant was obtained by the coefficient of determination (R^2^). For this purpose, each informant’s age was scored according to the following ranges: 1 (for 28–40), 2 (for 40–50), 3 (for 50–60), 4 (for 60–70) and 5 (for 70–81).

### Methodology proposed

The proposed method involves three steps. Preprocessing the data is the first step. This approach eliminates unnecessary features and simplifies them. By removing inconsistent features, predictions can be improved and execution times reduced. At this stage, data mining algorithms depend on uppercase letters, lowercase letters, text spaces, etc. Therefore, the data is integrated first. After that, one of the sample values of the "mode of application" column is empty, and from where the value of this column is empty. So, this sample is removed from the dataset. Also, some columns that have no role in the prediction process such as “Scientific name”, “Vernacular name (Persian)”, “Voucher no” are removed from the dataset.

In the second step, the preprocessed samples are divided into training and testing. In the current study, 70% of the data is used for training and 30% for testing. The 10-fold cross-validation method has been used to train the proposed model.

Once the data has been preprocessed and converted into an optimal dataset, it can be fed into different classification algorithms (supervised learning). To classify the dataset, support vector machines, J48 decision trees, neural networks, and logistic regression were employed. All data mining algorithms are tested on the dataset with different parameters.

**Fig 1:**
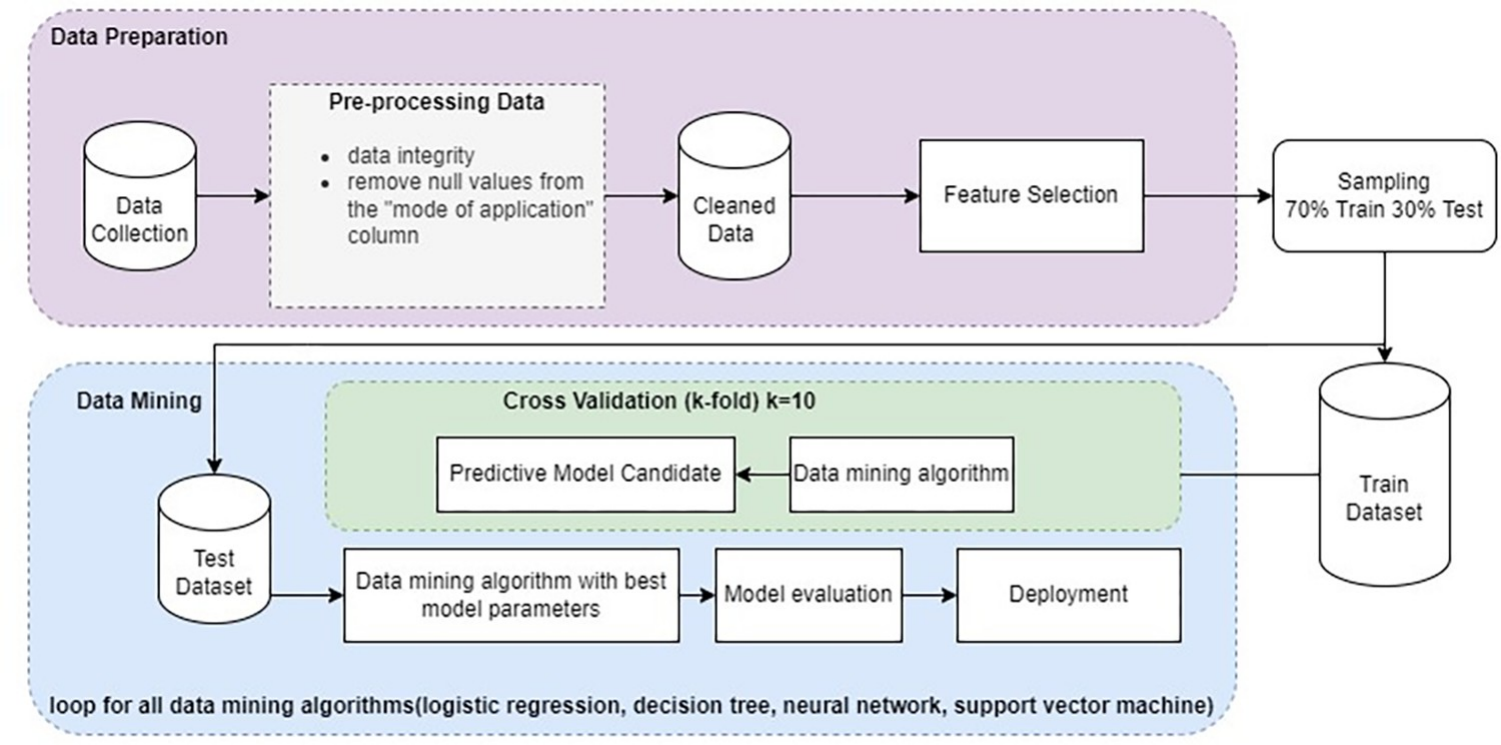
Flowchart of the proposed method.

In the third step, the proposed model is evaluated using various model evaluation criteria. Several criteria are used to evaluate the developed method, including F-measure, Recall, Precision, Receiver Operating Characteristic (ROC), and Cohen’s Kappa. Fig 1 illustrates the flowchart of the proposed method.

## Results and discussion

Shahrbabak region’s choice to study ethnopharmacology was determined for several reasons. Firstly, its historical significance as an ancient Iranian city believed to have been established by Sassanid king Ardeshir Babakan around 1800 years ago offers a rich cultural and historical context for ethnopharmacological research [34]. Additionally, this town is amidst a semi-arid climate and close to significant copper mines, influencing local ethnopharmacological practices and offering a rich cultural context for research. This study will significantly benefit researchers, scientists, herbal enthusiasts, and pharmaceutical professionals. Researchers can use data mining techniques to analyze and interpret traditional medicinal plant knowledge. A valuable resource has been provided to pharmaceutical researchers by the current study, allowing them to explore and develop novel drugs in the future. This contributes to pharmaceutical science advancement and healthcare solutions improvement.

### Plant diversity

**Fig 2:**
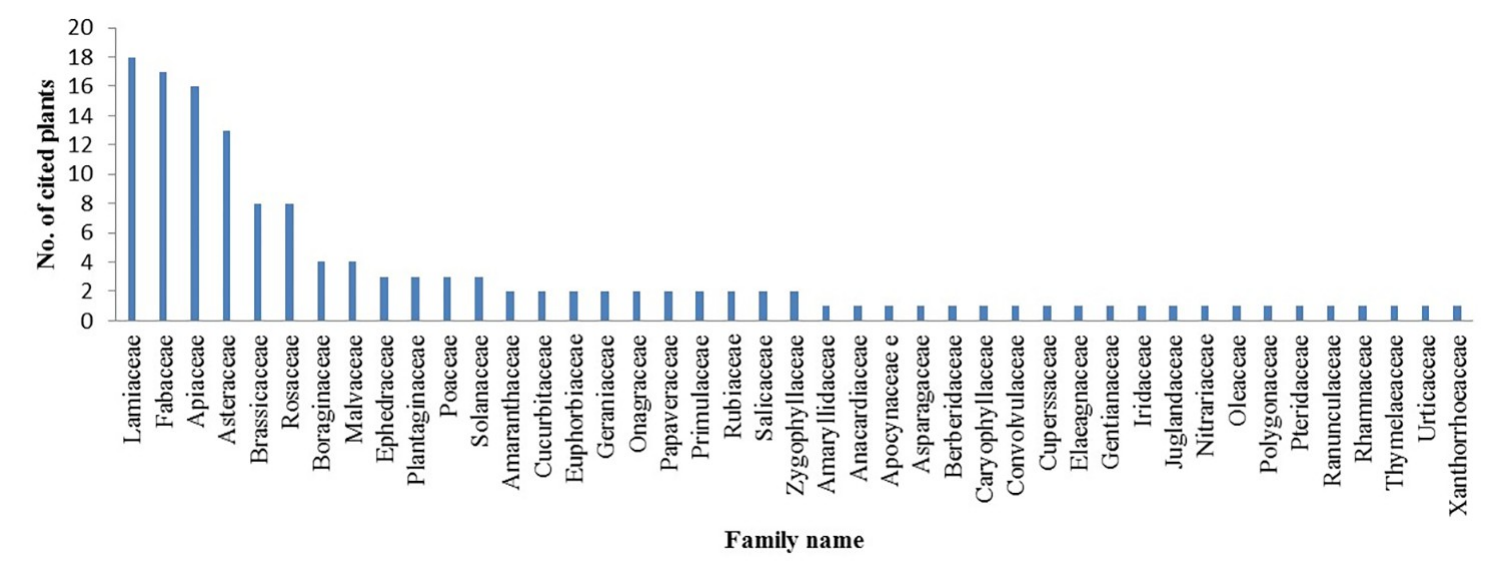
The number of medicinal species in each family.

All 141 plant species in this study were considered medicinal by indigenous people. These medicinal plants are from 43 different families, among which the Lamiaceae has 18 species, Fabaceae has 17 and Apiaceae has 16 species, which were found to be the most frequently occurring families among the 141 species in the area, followed by Asteraceae with 13 species (Fig 2). According to a previous report from Iran, the Lamiaceae and Apiaceae families have the highest number of medicinal plants in their local area. [16]. According to other studies, the Lamiaceae are the most abundant family of medicinal plants in the Kerman province [15]. Furthermore, the Lamiaceae family has plants with medicinal properties that enable their use as sources of traditional drugs. These can be applied to digestive disorders, menstrual disorders, hepatitis, and liver diseases [35,36].

### Plant parts are used as medicinal agents

**Fig 3:**
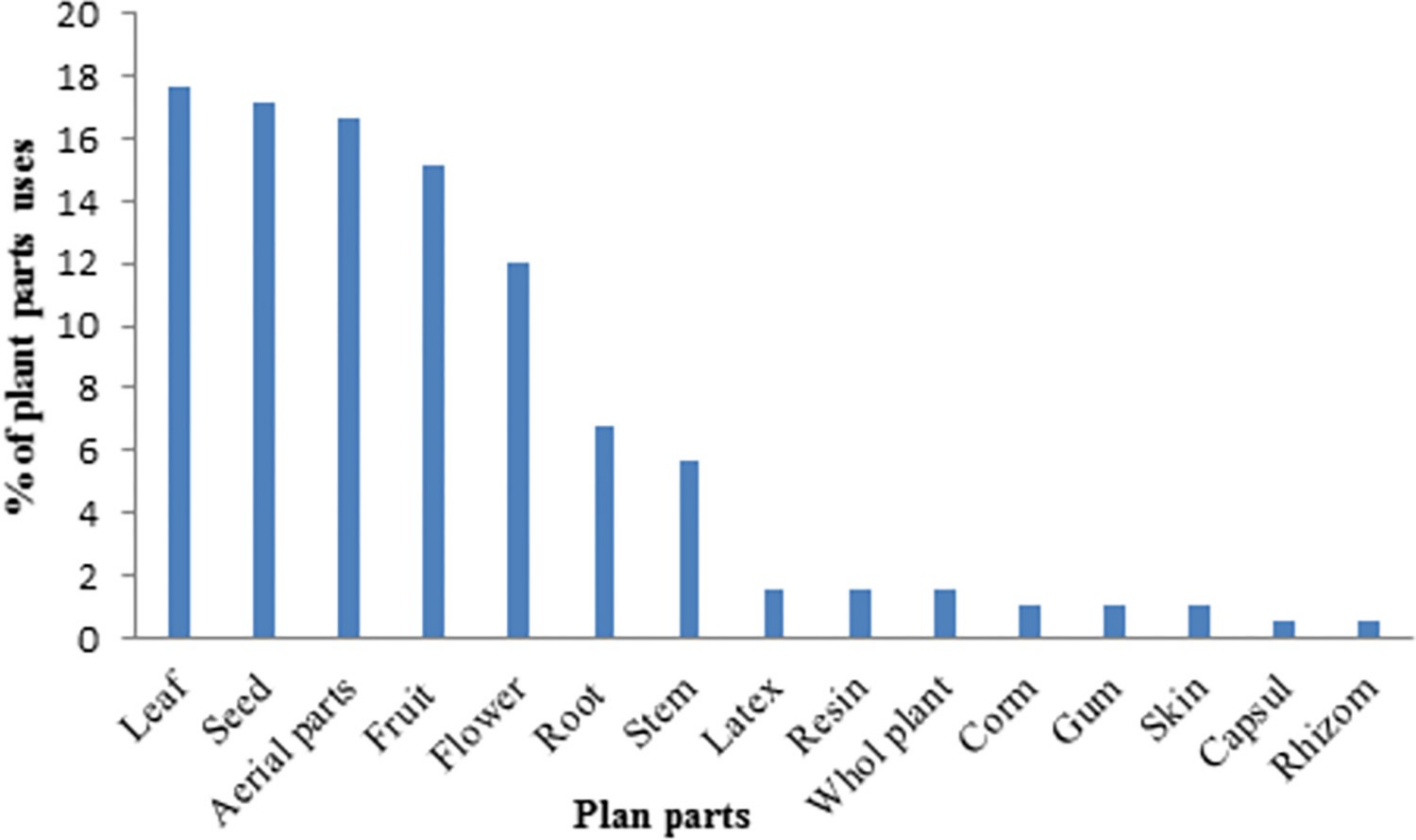
Percentage of used plant parts.

Local people reported using different plant parts. The most common parts used were leaf (17.7%), seed (17.1%), aerial parts (16.6%), fruit (15.1%) and flower (11.9%), respectively (Fig 3). In contrast, indigenous people were least likely to use whole plants, corns, gums, skin, capsules, and rhizomes of plants. The leaf was the most popular, which could be explained botanically by photosynthesis-producing compounds such as chlorophyll, flavonoids, alkaloids, and other bioactive molecules [37,38]. These results support previous reports that leaf, fruit, and aerial parts are mainly medicinal [12,16].

### Preparation and modes of application

**Fig 4:**
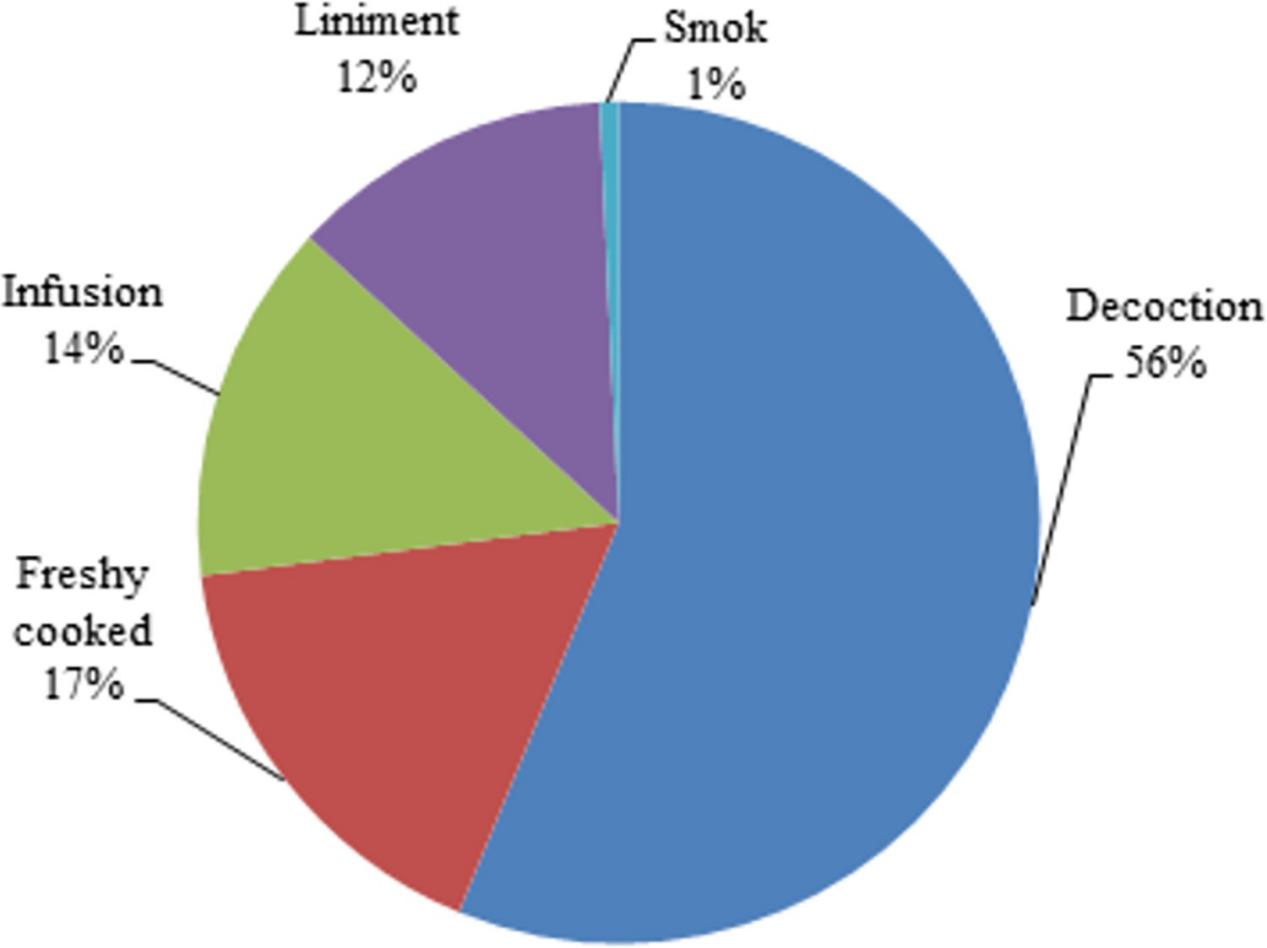
Modes of preparations used of medicinal plants for treatment.

The decoction was found to be the most frequently used method (56%) for preparing plant materials before medicine application. Other methods of preparing the plants were by processing them as freshly cooked (with a prevalence of 17%), using them by infusion (14%) and as liniment (12%) (Fig 4). Due to its ease of use, decoction is usually the most widely used method for medicinal plant preparation before consumption [39]. Use methods are oral, topical and combined. In the available literature, most plants are reported to be consumed orally, while the topical mode of application is subordinate to oral consumption (Table 1). For instance, *Centaurium pulchellum* subsp. *grandiflorum* (Batt.) Maire, *Geranium rotundifolium L*. and *Berberis jamesiana* Forrest & W.W.Sm.. are used only as oral, whereas *Ducrosia assadii* Alava. and *Cymbopogon schoenanthus* (L.) Spreng. are used by topical modes of application, and some species such as *Sanguisorba minor* Scop. and *Papaver dubium* L. are used orally and topically concurrently. Various methods are employed for plant preparation and application, emphasizing the diversity and complex nature of traditional medicinal practices. Traditional knowledge of medicinal plant use can be gained by understanding the various modes of application. This highlights the potential and adaptability of these natural resources to address healthcare needs [40].

### The life cycle of plants

**Fig 5:**
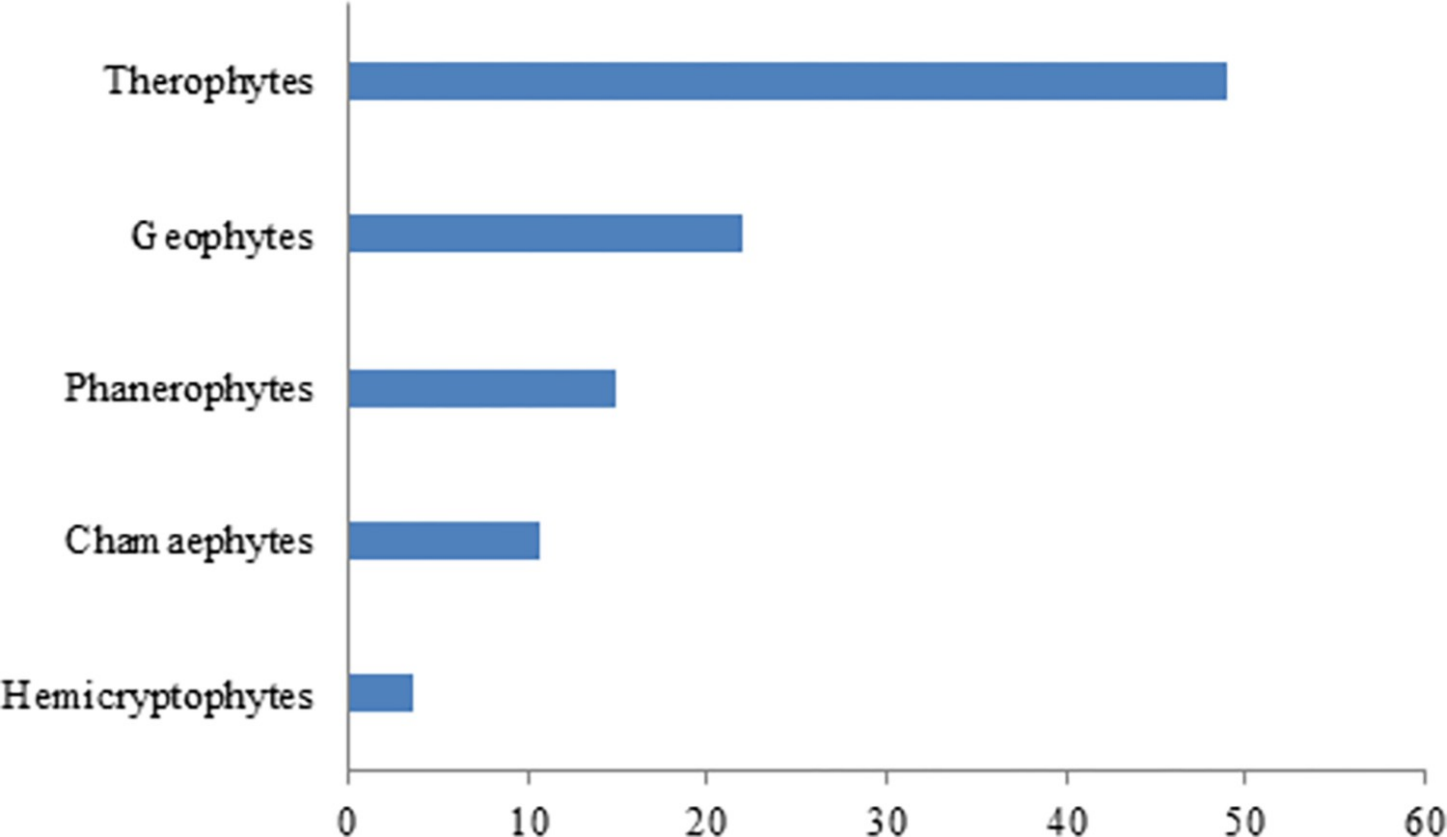
Life grown forms of wild medicinal species from Shahrbābak area.

An analysis of the life cycles shows the dominance of Therophytes (48.93%) and Geophytes (21.98%) in the flora species in the current study (Fig 5). Geophytes and Therophytes in a region can provide valuable information about the availability and seasonal variations of medicinal plants used by local communities [41]. As a result of Therophytes’ ability to grow in adverse conditions and germinate quickly after rain, medicinal plant resources may be abundant during certain seasons [42]. Additionally, Geophytes can store vital nutrients underground, ensuring a continuous supply of medicinal plants, especially in arid climates [43].

### Records and categories

**Fig 6:**
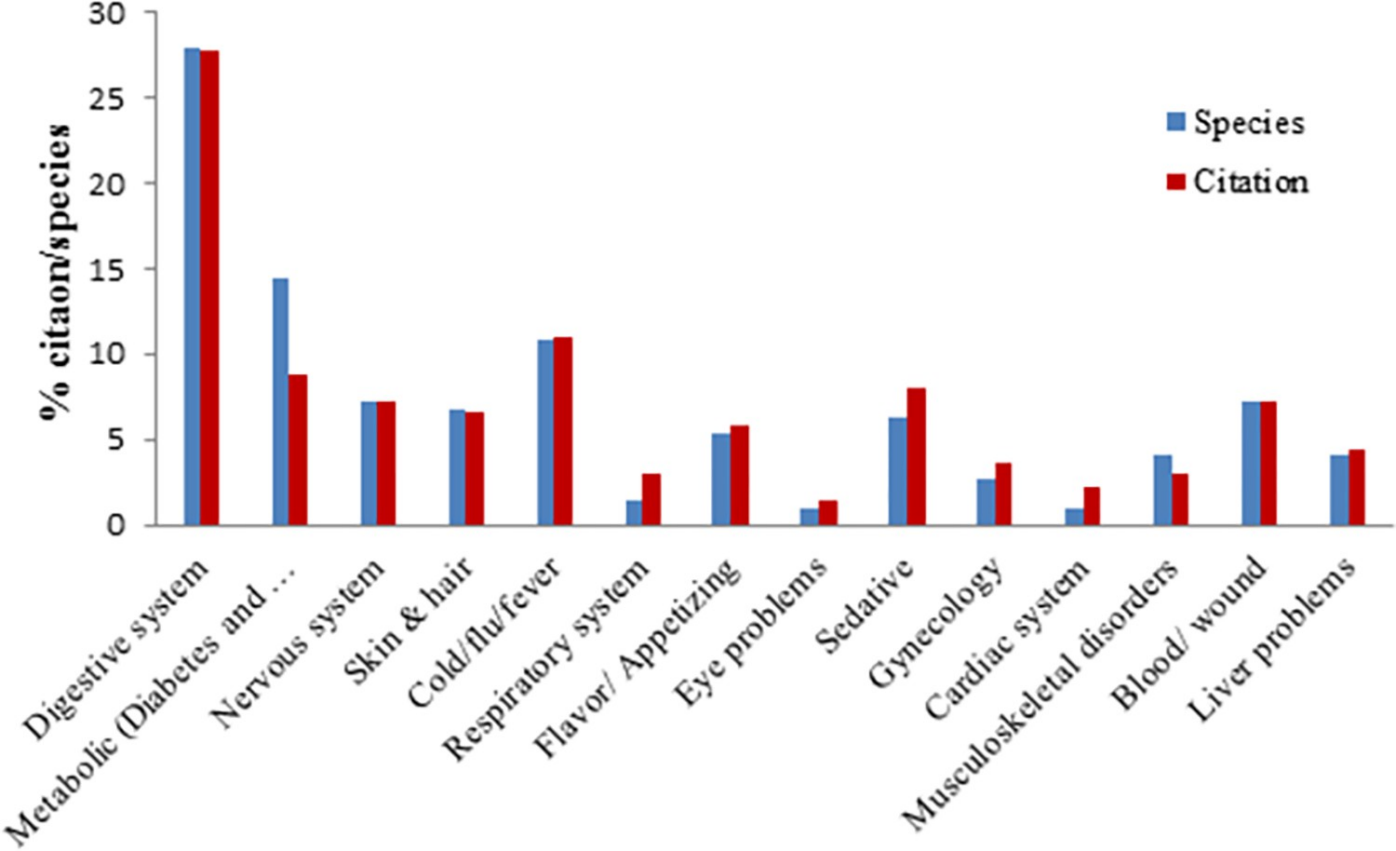
Percentage of species and citation in each medicinal use category.

Based on the data collected from the informants, a total number of 222 medicinal applications are reported in this work, which can be categorized into 14 groups which heal disorders of the digestive system (27.92%) as the most common ailment treated by plants, followed by metabolic disorders (14.41%), cold-flu and fever (10.81%), and problems of the nervous system (7.2%) (Fig 6). These results are similar to other studies in which many medicinal plants were used to alleviate digestive disorders [9,10,12,15,16,44]. Among these categories, some other ailments, such as constipation, diarrhea, and influenza, have been commonly treated in the Shahrbabak.

### Comparison of different indices

Table 2 presents the results obtained from the ICF values for the categorized ailments. Metabolic (0.64) and musculoskeletal disorders (0.62) had the highest ICF value and included ailments such as kidney stones, urinary infections, diuretics and rheumatism, headaches and skeletal fractures. Also, the respiratory system (0.5) had a high ICF value and was followed by skin and hair (0.43), digestive system (0.4), nervous system (0.4), cold/flu/fever (0.4) and cuts/wounds (0.4). For liver problems and flavor/appetizing, the ICF values were 0.37 and 0.36, respectively. When the ICF index is very low, informants do not exchange extensive amounts of information about the use of species to treat diseases [11]. The digestive system was claimed to be treated most commonly (with 20 plants), followed by cold/flu/fever (with 15 plants), sedative ailments (with 11 plants), the nervous system (with ten plants), cuts/wounds (with ten plants) and skin and hair (with nine plants). According to the current study, metabolic and musculoskeletal disorders are the most common ailments in the Shahrbabak region. The current findings seem to be consistent with other research findings which found that metabolic disorders had the highest ICF in Sirjan, a city in the Kerman province [15] and Rasuwa District in Central Nepal [45]. Nonetheless, these results differ from some other published reports from Iran such as those carried out in the south of Kerman [16] and in the Kohgiluyeh and Boyer Ahmad provinces [46].

**Table 2:** ICF and diseases based categories.

Use category	Use citation	No. of plant used	Category uses taxon ICF
Digestive system	62	38	0.40
Metabolic (Diabetes and diuretic)	32	12	0.64
Nervous system	16	10	0.4
Skin & hair	15	9	0.43
Cold/flu/fever	24	15	0.40
Respiratory system	3	4	0.50
Flavor/ Appetizing	12	8	0.36
Eye problems	2	2	-
Sedative	14	11	0.23
Gynecology	6	5	0.20
Cardiac system	2	3	-
Musculoskeletal disorders	9	4	0.62
Blood/ wound	16	10	0.40
Liver problems	9	6	0.37

**Fig 7:**
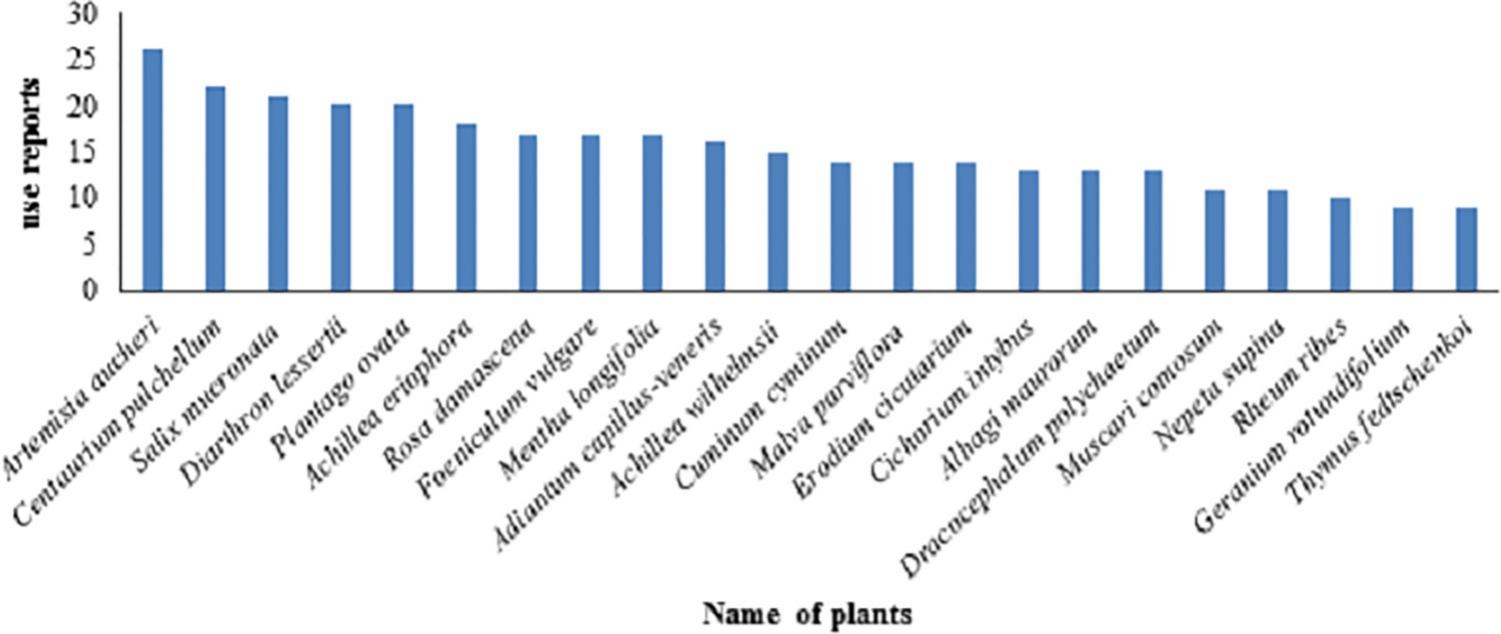
Plants with the highest number of use report.

*Adiantum capillus-veneris* L. and *Plantago ovata* are the most prized plants in this region, therefore many informants confirmed that these are useful plants (Table 3). The number of informants reporting a specific use for a plant species is called a ‘Use Report’ (UR). *Artemisia aucheri* had the maximum number of reports confirming its medicinal use (26 UR), followed by *Centaurium pulchellum (22 UR)*, *Salix mucronata* Thunb. (21 UR), *Diarthron lessertii* (Wikstr.) Kit Tan. and *Plantago ovata* with (20 UR) (Fig 7). Sadat-Hosseini et al reported that *Chrysanthemum parthenium* (L.) Pers. and *Cerasus mahaleb* (L.) Mill. had the highest number of uses, reasserting their medicinal purpose (23) in the south of Kerman. [16]. Nasab and Khosravi studied the Sirjan region in Kerman and discovered *Malva sylvestris* L. has the highest number of medicinal use reports [15].

**Table 3:** Comparison of important medicinal plants by using indices and species ranking based on each index.

Family	Scientific name	RFC	CI	RFC ranking	CI ranking
*Pteridaceae*	*Adianthum capillus-veneris*	0.6666	0.7619	1	7
*lantaginaceae*	*Plantago ovata*	0.6666	0.9523	1	4
*Malvaceae*	*Malva microcarpa*	0.5714	0.6666	2	9
*Asteraceae*	*Achillea eriophora*	0.4761	0.8571	3	5
*Asteraceae*	*Artemisia aucheri*	0.4285	1.2380	4	1
*Salicaceae*	*Salix mucronata *	0.3333	1	5	3
*Lamiaceae*	*Thymus fedtschenkoi*	0.3333	0.4285	5	13
*Geraniaceae*	*Erodium cicutarium*	0.2857	0.6666	6	9
*Lamiaceae*	*Nepeta supina*	0.2857	0.5238	6	11
*Fabaceae*	*Alhagi maurorum*	0.2380	0.6190	7	10
*Lamiaceae*	*Mentha longifolia*	0.2380	0.8095	7	6
Apiaceae	*Cuminum cyminum*	0.1904	0.6666	8	9
*Gentianaceae*	*Centaurium pulchellum*	0.1428	1.0476	9	2
*Thymelaeaceae*	*Diarthron lessertii*	0.1428	0.9523	9	4
Apiaceae	*Foeniculum vulgare*	0.1428	0.8095	9	6
*Rosaceae*	*Rosa damascena*	0.1428	0.8095	9	6
*Asteraceae*	*Achillea santolinoides*	0.0952	0.7142	10	8
*Asteraceae*	*Cichorium intybus*	0.0952	0.6190	10	10
*Lamiaceae*	*Dracocephalum polychaetum*	0.0952	0.6190	10	10
*Geraniaceae*	*Geranium rotundifolium*	0.0952	0.4285	10	13
*Asparagacea*	*Muscari comosum *	0.0952	0.5231	10	11
*Polygonaceae*	*Rheum ribes*	0.0952	0.4761	10	12

RFC, ralative frequency of citation and CI, index of cultural importance.

**Fig 8:**
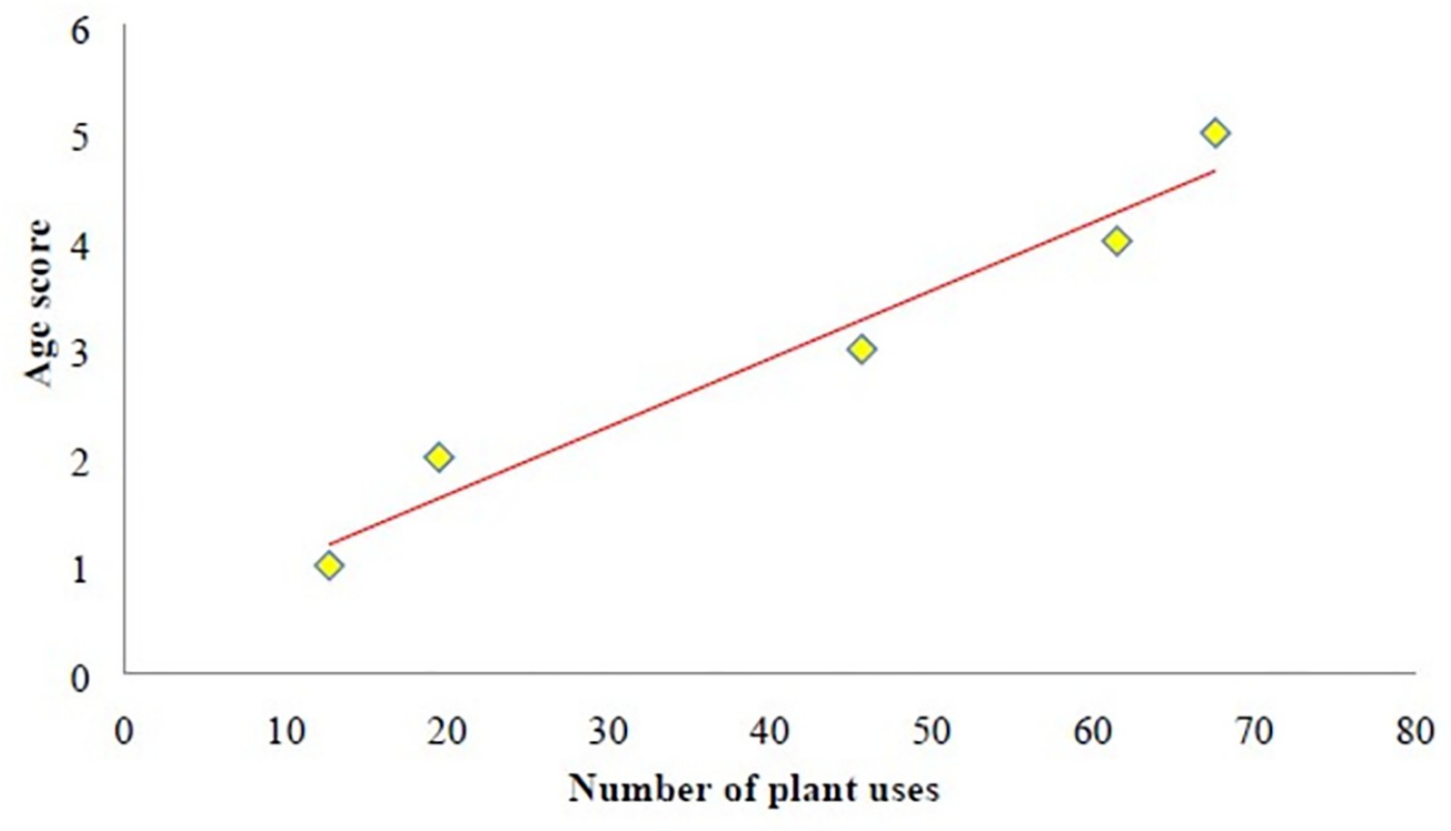
The liner regression model between informant’s age and number of plant species uses.

Table 3 provide the results obtained from the RFC and CI indices, respectively. The most critical species according to the RFC index are *A*. *capillus-veneris*, *P*. *ovata*,*Malva parviflora* var. *Parviflora*. and *Genista tinctoria* L. It can therefore be suggested that these species are commonly recognized by many informants in the Shahrbabak. However, *A*. *aucheri* ranked first through the CI index, and Table 3 shows the ranking based on CI and RFC indices. These results differ from some published studies. For instance, Sadat-Hosseini et al reported that *C*. *mahaleb* and *C*. *parthenium* ranked first in Kerman’s south [16], while Mosaddegh et al indicated that Teucrium *polium* L. ranked first in the Kohgiluyeh and Boyer Ahmad province [12]. The linear regression model drawn between informants’ age and the number of reported uses for a given medicinal plant is significant (P-value = 0.004; Fig 8). This indicates that older informants have more knowledge of the use of medicinal plants. RFC and CI indices show that the best-known plants have major chemical compounds (Table 4), such as 1,8-Cineol and α-pinene.

**Table 4:** Main compounds of importance of medicinal species.

Name of plants	Compounds	Reference
*Adianthum capillus-veneris*	α-D-Glucopyranoside, 5,7-Dodecadiyn -1 and 3-Trifluoroacetoxypentadecane	Hussein et al., (2016)
*Artemisia aucheri*	1,8-cineol, chrysanthenone, α-pinene and mesitylene	Hashemi et al., (2007)
*Centaurium pulchellum*	caryophyllene oxide, β-damascenone and β-ionone	Mihailović et al., (2011)
*Salix mucronata *	1,4-Dimethoxybenzene, phenylethyl alcohol, carvone and citronellol	Karimi et al., (2011)
*Achillea eriophora*	camphor, 1,8 cineol and camphene	Ghani et al., (2008)
*Thymus fedtschenkoi*	thymol, carvacrol, p-cymene and 1,8-cineole	Abousaber et al., (2002)
*Erodium cicutarium*	isomenthone, citronellol, geraniol and methyl eugenol	Lis-Balchin, (1993)
*Alhagi maurorum*	Oxygenated sesquiterpenes, Drimenol, 9-Octylheptadecane, Neophytadiene and Hydrocarbons	Samejo et al., (2012)
*Mentha longifolia*	Pulegone, isomenthone, 1,8-cineole, borneol, and piperitenone oxide	Mkaddem et al., (2009)
*Cuminum cyminum*	α-pinene, 1,8-cineole and linalool	Gachkar et al., (2007)
*Foeniculum vulgare*	trans-anethole, α-pinene, β-myrcene, limonene, and α-terpinene	Telci et al., (2009)
*Rosa damascena*	linalool, nerol, geraniol, 1-nonadecene, n-tricosane, hexatriacontane and n-pentacosane	Yassa et al., (2009)
*Achillea santolinoides*	Camphor, α-pinene and1,8-cineole	Sanz et al., (1985)
*Cichorium intybus*	Octane, n-nonadecane, pentadecanone and hexadecane	Judžentienė et al., (2008)
*Dracocephalum polychaetum*	Oxygenated, hydrocarbon, perilla aldehyde and limonene	Sonboli et al., (2012)
*Geranium rotundifolium*	a-terpinyl acetate and pulegone	Barazandeh (2004)
*Rheum ribes*	palmitic acid, n-eicosane, n-tetracosane, linoleic acid and ethyl linoleate	Naeimi et al., (2014)

### Medicinal plants are used in combinations

In some cases, indigenous people treated diseases using a combination of medicinal plants. For example, combining *Foeniculum vulgare* Mill., *Elwendia persica* (Boiss.) Pimenov & Kljuykov and *Cuminum cyminum* L. alleviated carminative and gastric discomforts. Also, the combination of *Tanacetum parthenium* (L.), *Ocimum basilicum* L. and *Nepeta glomerulosa* Boiss. was reported to be effective as a nerve tonic. Traditional medicine utilizes combined medicinal plants to enhance therapeutic and minimize side effects [47]. By using this approach, new treatment strategies can be developed and local plants can be identified for drug development [48].

### Side effects of medicinal plants

Informants believe combining *F*. *vulgare*, *B*. *persicum*, and *C*. *cyminum* can improve digestion. However, it can cause abortions in some women. *Ferula* species may also cause diarrhea in children and adults. Medicinal plants’ side effects are influenced by an individual’s reaction, dosage, preparation method, and interactions with other medications or health conditions [49,50]. Esmaeilzadeh, reported that herbal combinations can benefit certain ailments, but also present risks, such as abortion risk for women [49].

### Comparison of plants identified in the current study with previous studies

A comparison between this study and 14 similar studies (in Iran and other countries) was conducted to identify the plants that were reportedly medicinal in the current work for the first time in the available literature. Previous studies were carried out in various regions of Iran, including Sirjan [15], south of Kerman [16], Kohgiluyeh and Boyer Ahmad [12], Saravan [11], Turkmen Sahra [9] and West Azarbaijan [10]. Other countries in which studies have been conducted include Pakistan [51,52], Sri Lanka [53], Brazil [54], China [55], Morocco [56], Italy [57] and India [58]. Table 5 presents the results of the comparing medicinal plants with other reports. Following the literature review, 57 of the 141 species are reported here for the first time to have medicinal uses.

**Table 5:** Comparative presence-absence matrix for the recorded plant species.

Code	A	B	C	D	E	F	G	H	I	J	K	L	M	N	Code	A	B	C	D	E	F	G	H	I	J	K	L	M	N
**1**	0	0	0	0	0	0	0	0	0	0	0	0	0	0	73	0	1	0	0	0	0	0	0	0	0	0	0	0	0
**2**	0	0	0	0	0	0	0	0	0	0	0	0	0	0	74	0	0	1	0	1	1	0	0	0	0	0	1	0	1
3	0	1	0	0	0	0	0	0	0	0	0	0	0	0	**75**	0	0	0	0	0	0	0	0	0	0	0	0	0	0
4	0	1	0	1	0	0	0	0	0	0	0	0	0	0	**76**	0	0	0	0	0	0	0	0	0	0	0	0	0	0
5	1	1	0	0	1	0	0	0	0	0	0	0	1	0	**77**	0	0	0	0	0	0	0	0	0	0	0	0	0	0
6	1	1	0	0	0	0	0	0	0	0	0	0	0	0	**78**	0	0	0	0	0	0	0	0	0	0	0	0	0	0
7	0	0	0	1	0	0	0	0	0	0	0	1	0	0	**79**	0	0	0	0	0	0	0	0	0	0	0	0	0	0
8	1	0	0	0	0	0	0	0	0	0	0	0	0	1	80	0	0	1	0	0	0	0	0	0	0	0	0	0	0
9	1	1	0	0	1	0	1	0	1	0	0	0	0	0	**81**	0	0	0	0	0	0	0	0	0	0	0	0	0	0
10	0	0	0	0	0	0	0	0	0	0	0	0	0	1	**82**	0	0	0	0	0	0	0	0	0	0	0	0	0	0
11	0	0	0	1	0	0	0	0	0	0	0	0	0	0	**83**	0	0	0	0	0	0	0	0	0	0	0	0	0	0
**12**	0	0	0	0	0	0	0	0	0	0	0	0	0	0	84	0	1	0	0	0	0	0	0	0	0	0	0	0	0
13	0	0	0	1	0	0	0	0	0	0	0	0	0	0	85	1	1	0	0	0	0	0	0	0	0	0	0	0	0
**14**	0	0	0	0	0	0	0	0	0	0	0	0	0	0	86	1	1	0	0	0	0	1	1	0	0	0	0	0	0
**15**	0	0	0	0	0	0	0	0	0	0	0	0	0	0	87	0	1	0	0	0	0	0	0	0	0	0	0	0	0
16	0	0	1	0	0	0	0	0	0	0	0	0	0	0	**88**	0	0	0	0	0	0	0	0	0	0	0	0	0	0
17	0	1	0	0	0	0	0	0	0	0	0	0	0	0	**89**	0	0	0	0	0	0	0	0	0	0	0	0	0	0
18	1	1	1	1	1	0	1	0	0	0	0	1	1	0	90	0	0	1	0	0	0	0	0	0	0	0	0	0	0
19	0	0	0	0	0	0	0	0	0	0	0	0	0	0	91	0	0	0	0	0	0	1	0	0	1	0	0	1	0
20	0	1	0	0	0	0	0	0	0	0	0	0	0	0	**92**	0	0	0	0	0	0	0	0	0	0	0	0	0	0
21	0	0	0	0	0	0	1	0	1	0	0	1	0	0	93	0	1	0	0	0	0	0	0	0	0	0	0	0	0
**22**	0	0	0	0	0	0	0	0	0	0	0	0	0	0	94	1	1	1	1	1	0	0	0	0	0	0	1	0	0
23	0	1	0	0	0	0	0	0	0	0	0	0	0	0	**95**	0	0	0	0	0	0	0	0	0	0	0	0	0	0
**24**	0	0	0	0	0	0	0	0	0	0	0	0	0	0	96	0	1	1	0	1	0	0	0	0	0	0	0	0	0
25	0	1	0	0	0	0	0	0	0	0	0	0	0	0	97	1	0	0	0	0	0	0	0	0	0	0	0	0	0
26	0	1	0	0	0	0	0	0	0	0	0	0	0	0	98	1	0	0	1	0	0	0	0	0	0	0	0	1	1
27	0	0	1	0	0	1	1	0	0	0	0	1	0	0	**99**	0	0	0	0	0	0	0	0	0	0	0	0	0	0
28	0	1	0	0	0	0	0	0	0	0	0	0	0	0	100	0	1	0	0	0	0	0	0	0	0	0	0	0	0
**29**	0	0	0	0	0	0	0	0	0	0	0	0	0	0	**101**	0	0	0	0	0	0	0	0	0	0	0	0	0	0
30	0	0	1	0	0	0	0	0	0	0	0	0	0	0	102	0	0	0	0	0	0	0	1	0	0	1	0	0	0
31	0	1	0	0	0	0	0	0	0	0	0	0	0	0	103	0	0	0	0	0	0	0	0	0	1	0	0	0	0
**32**	0	0	0	0	0	0	0	0	0	0	0	0	0	0	104	1	0	1	1	1	0	0	0	0	0	0	0	0	0
33	0	1	0	0	0	0	0	0	0	0	0	0	0	0	105	0	0	0	0	0	0	0	0	1	0	0	0	0	0
**34**	0	0	0	0	0	0	0	0	0	0	0	0	0	0	**106**	0	0	0	0	0	0	0	0	0	0	0	0	0	0
35	0	1	0	0	0	0	0	0	0	0	0	0	0	0	**107**	0	0	0	0	0	0	0	0	0	0	0	0	0	0
36	1	1	1	0	0	0	0	0	0	0	0	0	0	0	108	1	1	1	0	0	0	0	0	0	0	0	0	0	0
37	0	0	0	0	1	0	0	0	0	0	0	0	0	0	**109**	0	0	0	0	0	0	0	0	0	0	0	0	0	0
38	0	0	0	0	1	0	0	0	0	0	0	0	0	0	110	1	0	0	0	1	0	0	0	0	0	0	0	1	0
**39**	0	0	0	0	0	0	0	0	0	0	0	0	0	0	111	1	0	0	0	0	1	0	0	0	0	0	0	0	0
40	1	1	1	1	1	0	0	0	0	0	0	0	0	0	**112**	0	0	0	0	0	0	0	0	0	0	0	0	0	0
**41**	0	0	0	0	0	0	0	0	0	0	0	0	0	0	**113**	0	0	0	0	0	0	0	0	0	0	0	0	0	0
42	0	0	1	0	0	1	0	0	0	0	0	0	0	0	**114**	0	0	0	0	0	0	0	0	0	0	0	0	0	0
43	1	1	1	1	1	0	0	0	0	0	0	0	0	0	115	0	0	0	0	0	0	0	0	0	0	1	0	0	0
**44**	0	0	0	0	0	0	0	0	0	0	0	0	0	0	116	0	0	1	0	0	0	0	0	0	0	0	0	0	0
45	0	0	1	0	0	0	0	0	0	0	0	0	0	0	**117**	0	0	0	0	0	0	0	0	0	0	0	0	0	0
**46**	0	0	0	0	0	0	0	0	0	0	0	0	0	0	**118**	0	0	0	0	0	0	0	0	0	0	0	0	0	0
**47**	0	0	0	0	0	0	0	0	0	0	0	0	0	0	119	0	0	1	0	1	0	0	0	0	0	0	0	0	0
48	0	1	0	0	0	0	0	0	0	0	0	0	0	0	**120**	0	0	0	0	0	0	0	0	0	0	0	0	0	0
**49**	0	0	0	0	0	0	0	0	0	0	0	0	0	0	121	0	0	1	0	0	0	0	0	0	0	0	0	0	0
50	0	1	0	0	0	0	0	0	0	0	0	0	0	0	122	0	1	0	0	0	0	0	0	0	0	0	0	0	0
51	0	1	1	0	0	0	0	0	0	0	1	0	0	0	123	0	1	1	0	0	0	0	0	0	0	0	0	0	0
52	0	0	0	0	0	0	0	0	0	1	0	0	0	0	**124**	0	0	0	0	0	0	0	0	0	0	0	0	0	0
53	0	0	1	0	0	0	0	0	0	0	0	0	0	0	**125**	0	0	0	0	0	0	0	0	0	0	0	0	0	0
54	1	0	0	0	0	0	0	0	0	0	0	0	0	0	126	0	0	1	0	1	1	0	0	0	0	0	0	1	0
**55**	0	0	0	0	0	0	0	0	0	0	0	0	0	0	127	0	0	1	0	0	1	0	0	0	0	0	0	0	0
**56**	0	0	0	0	0	0	0	0	0	0	0	0	0	0	**128**	0	0	0	0	0	0	0	0	0	0	0	0	0	0
57	1	1	0	0	0	0	1	1	0	0	0	0	0	0	129	0	1	1	0	0	0	0	0	0	0	0	0	0	0
58	0	0	0	1	0	0	0	0	0	0	0	0	0	0	**130**	0	0	0	0	0	0	0	0	0	0	0	0	0	0
59	0	1	1	0	0	0	0	0	1	1	0	0	0	1	**131**	0	0	0	0	0	0	0	0	0	0	0	0	0	0
60	0	0	0	0	1	0	0	0	0	0	0	0	0	0	**132**	0	0	0	0	0	0	0	0	0	0	0	0	0	0
61	1	0	1	0	0	0	0	0	0	0	0	0	0	0	**133**	0	0	0	0	0	0	0	0	0	0	0	0	0	0
62	0	1	0	0	0	0	0	0	0	0	0	0	0	0	134	0	0	0	1	0	1	0	0	0	0	0	0	0	0
**63**	0	0	0	0	0	0	0	0	0	0	0	0	0	0	135	0	0	1	0	0	0	0	0	0	0	0	0	0	0
**64**	0	0	0	0	0	0	0	0	0	0	0	0	0	0	**136**	0	0	0	0	0	0	0	0	0	0	0	0	0	0
**65**	0	0	0	0	0	0	0	0	0	0	0	0	0	0	**137**	0	0	0	0	0	0	0	0	0	0	0	0	0	0
66	0	1	0	0	0	0	0	0	0	0	0	0	0	0	**138**	0	0	0	0	0	0	0	0	0	0	0	0	0	0
67	1	1	1	0	1	1	0	0	1	0	0	0	0	0	139	0	0	1	0	0	0	0	0	0	0	0	0	0	0
68	1	1	0	0	1	0	0	0	0	0	0	0	0	0	140	0	0	0	1	1	0	0	0	0	0	0	0	0	0
**69**	0	0	0	0	0	0	0	0	0	0	0	0	0	0	141	0	0	0	0	1	0	0	0	0	0	0	0	0	0
70	0	1	0	0	0	0	0	0	0	0	0	0	0	0
**71**	0	0	0	0	0	0	0	0	0	0	0	0	0	0
**72**	0	0	0	0	0	0	0	0	0	0	0	0	0	0

A: (Nasab and Khosravi, 2014 [15]); B: (Sadat-Hosseini et al., 2017 [16]); C: (Mosaddegh et al., 2012 [12]); D: (Sadeghi et al., 2014 [11]); E: (Ghorbani, 2005 [9]); F: (Miraldi et al., 2001 [10]); G: (Ahmad et al., 2015 [5]); H: (Ishtiaq et al., 2015 [52]); I: (Dharmadasa et al., 2016 [53]); J: (Ribeiro et al., 2017 [54]); K: (Li et al., 2017 [55]); L: (Barkaoui et al., 2017 [56]); M: (Fortini et al., 2016 [57]); N: (Adhikari et al., 2018). Bold number not quoted in (A-N).

Other results from this comparison showed that some plants had a more comprehensive distribution range but different uses. In various studies, for example, *F*. *vulgare* is reported to have other uses for the treatment of various ailments such as abdominal pain and bloating [15], gastric discomfort, bone and joint pain [16], diuretic problems and kidney malfunctions [12]. It could also be used to treat menstrual disorders, or as a lactiferous agent. It could be used to alleviate coughs, asthma and digestive disorders, while also serving as a nerve tonic [11]. It is a carminative and hypnotic agent [9] and could treat hypertension [51], diabetes [56] and stomachache [57].

### Criteria for evaluation

**Fig 9:**
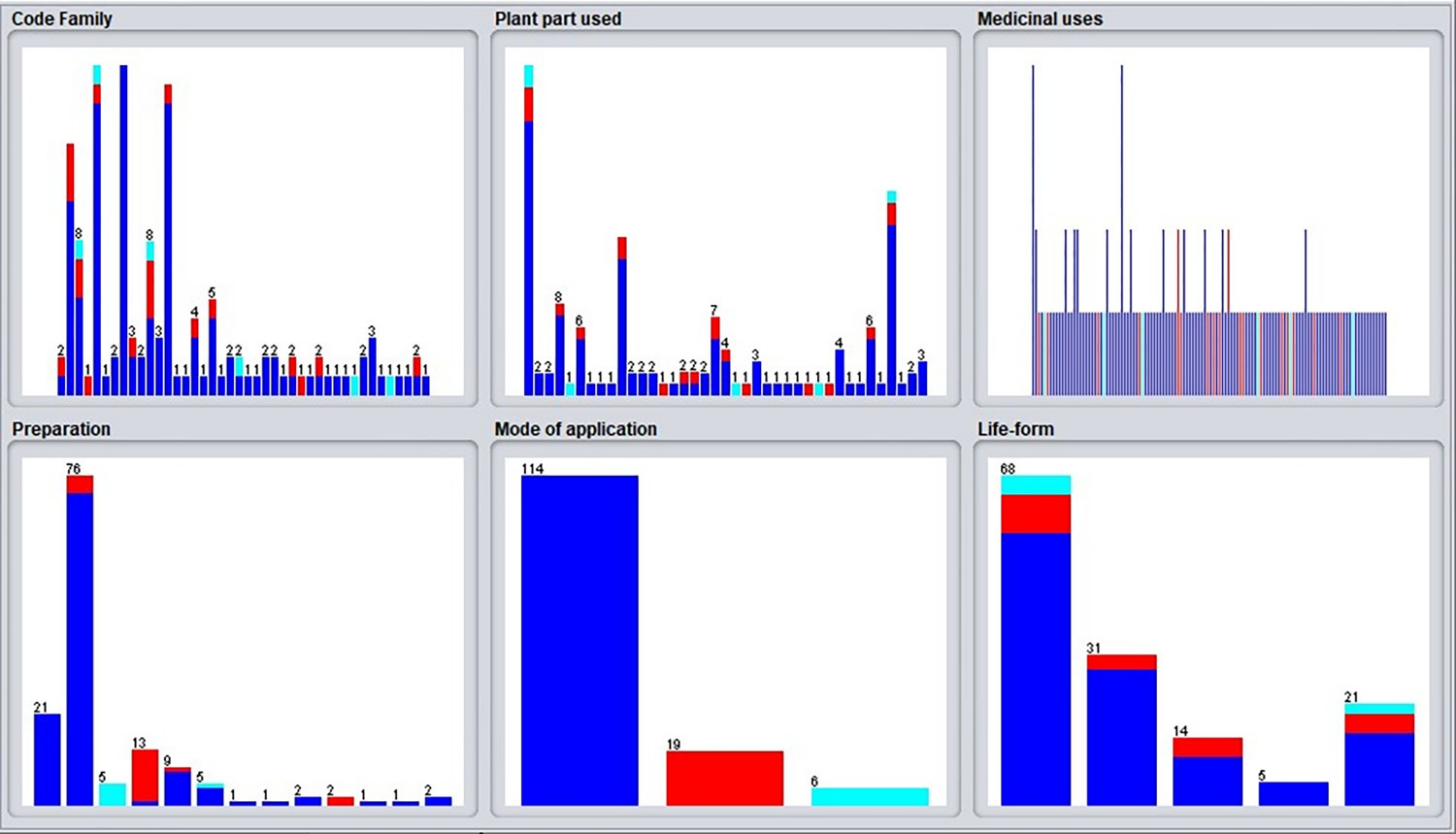
The amount of dispersion of samples of each class based on the class of "mode of application".

F-measure, Recall, Precision, Receiver Operating Characteristic (ROC) and Cohen’s Kappa are used in this study to evaluate the performance of the proposed method [59]. An important evaluation criterion in data mining is accuracy. Several studies have discovered the use of different assessment metrics in predictive modeling for medicinal plant uses. While accuracy is a vital criterion, it is significant to consider other metrics such as precision, recall, F-measure, Cohen’s Kappa coefficient, and ROC analysis [60–62]. These metrics can provide a more comprehensive understanding of model performance, particularly in multi-class classification problems. However, the choice of evaluation metric should be tailored to the specific objectives of the model, with accuracy being less suitable for particular applications [62]. It is possible to determine whether the proposed method accurately predicted the output based on accuracy. To obtain detailed information about the model, other evaluation criteria should be used besides measuring accuracy. The goal is to predict the mode of application of the medicinal plant, which includes three modes: oral, topical, or both. Based on the class of feature (mode of application), Fig 9 shows the distribution of each feature sample.

Table 6 presents the confusion matrix, the values can be in one of the categories TN (True Negative), TP (True Positive), FN (False Negative) and FP (False Positive).

- **TP:** The algorithm classified the sample in the positive category and the sample is also positive.
- **FP:** The algorithm classified the sample in the positive category, but the sample is negative.
- **TN:** The algorithm classified the sample in the negative category and the sample was also negative.
- **FN:** The algorithm classified the sample in the negative category, but the sample is positive.

**Table 6:** Confusion matrix.

		Actual values
		Positive	Negative
Predicted values	Positive	TP	FP
	Negative	FN	TN

In other words, when the algorithm mispredicts the sample class, the result will be FN or FP. When the algorithm correctly predicts the sample class, the result will be TN or TP. By using the following ratio, we can determine the model’s accuracy.

A model’s accuracy is determined by its ability to detect the medicinal plant’s mode of application correctly. The amount of data that can be recognized correctly equals the total number of available data. A model with a higher detection accuracy value will be more accurate and reliable. Eq (1) shows the accuracy evaluation criteria.


$$\mathrm{Accuracy}=\frac{\mathrm{TP}+\mathrm{TN}}{\mathrm{TP}+\mathrm{FN}+\mathrm{FP}+\mathrm{TN}}$$ (1)

### Precision

This evaluation criterion is used when the proposed method positively predicts the outcome. The precision criterion will be appropriate when the False Positive (FP) class detection accuracy value is high. Criteria for evaluating precision are given in relation (2).


$$\mathrm{Precision}=\frac{\mathrm{TP}}{\mathrm{TP}+\mathrm{FP}}$$ (2)

### Recall

The recall criteria are used to evaluate negative class detection accuracy. It is appropriate to use the Recall criterion when the false negative value (FN) is high. The Recall criterion is shown in Eq (3).


$$\mathrm{recall}=\frac{\mathrm{TP}}{\mathrm{TP}+\mathrm{FN}}$$ (3)

### F-measure

A critical evaluation criterion for model accuracy is the F-measure. The two measures of Recall and Precision are combined to form this criterion. Eq (4) shows the F-measure criterion.


F‐Measure=2*Precision*RecallPrecision+Recall (4)

### Cohen’s kappa coefficient

Cohen’s kappa coefficient is a numerical measure between -1 and +1, any measure closer to +1 indicates adequate performance, and the closer this value is to -1, it indicates disagreement. Cohen’s kappa coefficient is given in Eq (5).


$$K=\frac{P_{0-}P_{C}}{1-P_{C}}$$ (5)

### Receiver Operating Characteristic (ROC)

shows the area under the curve (AUC). A ROC analysis is one of the most critical evaluation criteria for supervised learning models. We can create a ROC curve by plotting the True Positive Rate against the False Positive Rate. Since the threshold is variable, a continuous graph will result.

### 10-fold cross-validation

**Fig 10:**
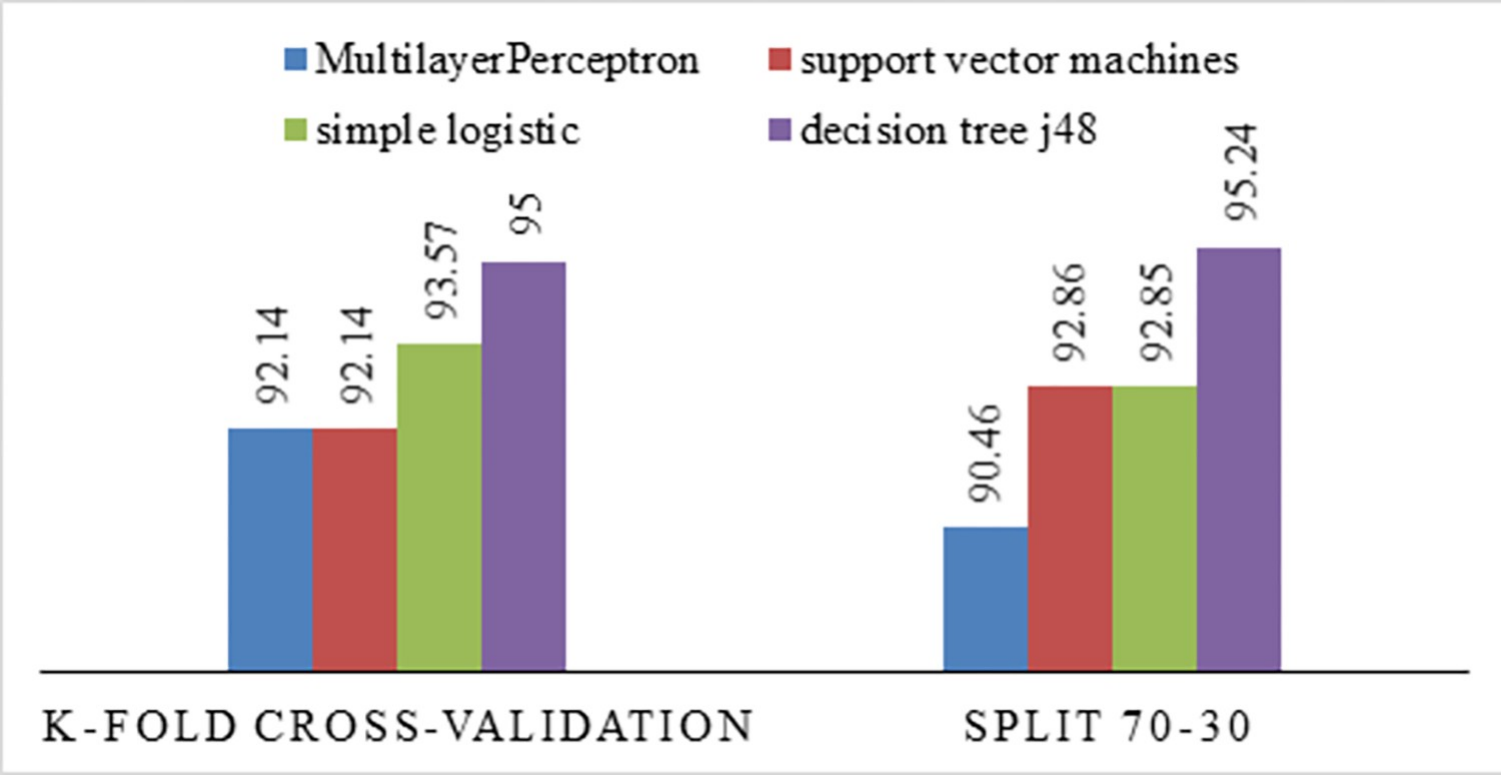
Comparison between accuracy through model training based on cross-validation and 70–30 split.

The K-fold cross-validation method proves the model’s performance. The 10-fold cross-validation method divides the original sample into ten equal parts. In each iteration, nine parts are considered training data, and one part is considered test data until the entire data is scrolled. In this method, the presented model was trained and tested ten times, and the result is an average accuracy of ten times. The benefit of using this approach is that it mitigates the overfitting risks linked to random sampling [59]. Fig 10 shows the test accuracy of different classification algorithms under the 70–30 split and 10-fold cross-validation. In the 70–30 split, 70% of the data was utilized for training the proposed model and 30% for testing the proposed model. The results show that in the 70–30 split, the J48 decision tree algorithm correctly predicted the dataset samples with an accuracy of 95.24%. In the 10-fold cross-validation, the J48 decision tree algorithm correctly assigned new samples to their respective classes with 95% accuracy. Since the 10-fold cross-validation is the average of ten times, and the number of dataset records is small, we use the 10-fold cross-validation method for prediction. Based on Fig 10, there is not much difference between 10-fold cross-validation and 30–70 division, and since the J48 decision-tree algorithm achieved 95% accuracy with cross-validation, this model is used. The confusion matrix table was used to calculate the model value based on different evaluation metrics. Table 7 shows that the J48 decision-tree algorithm achieved high accuracy in each evaluation metric, indicating that it is a very accurate algorithm. It is also more efficient than other algorithms.

**Table 7:** Comparison of evaluation criteria of the proposed model.

Algorithms criteria	J48 decision tree	Support vector machine	neural network	logistic regression
Precision	**95**	92	92	93.6
Recall	**95**	92.1	92.1	93.6
F-Measure	**94.8**	91.6	91.6	93.1
ROC Area	82.6	81.7	**83.1**	78.8
Kappa statistic	**82.13**	70.83	70.83	76.19

## Conclusion

Using data mining analysis, we gained valuable knowledge about medicinal plants uses. We noticed clear preferences for specific plant families, including Lamiaceae, Fabaceae, and Apiaceae, which are strongly inclined to apply leaves to medicinal preparations. Based on the current study results, the following conclusions and suggestions are presented:

- Focusing on documenting, standardizing, and preserving traditional knowledge and quality is crucial. We must carefully evaluate herbal combinations for potential side effects and consider dosage regulation and individual responses.
- For better reproducibility and understanding of ethnopharmacological studies, it should be considered that differences in language dialects and cultural interpretations could have influenced the data collection process and introduced complexities in data interpretation. Additionally, ensuring data quality from local informants raises concerns about reliability.
- Furthermore, predictive modeling based on machine learning algorithms showed promise for predicting plant applications. However, future works will be challenged by limited data availability, model generalization across diverse regions, and indigenous knowledge conservation and utilization.
- Future studies could investigate these medicinal plants’ compounds and biochemical properties using data mining algorithms. This scientific investigation could help identify their antibacterial, antifungal, antitoxic, or neutral properties.

While data mining provides valuable insight into medicinal plant usage, future studies should focus on standardization, ethical considerations, and strong model development.

## Supporting information

S1 Data Excel file figs 2, 3, 4, 5, 6, 7 and 10. (XLSX)

S1 File (DOCX)

## References

[1] Silambarasan R, Ayyanar M. An ethnobotanical study of medicinal plants in Palamalai region of Eastern Ghats, India. Journal of ethnopharmacology. 2015; 22:172:162–78. doi: 10.1016/j.jep.2015.05.046 26068426

[2] Houghton PJ. The role of plants in traditional medicine and current therapy. The Journal of Alternative and Complementary Medicine. 1995 Jun 1;1(2):131–43. doi: 10.1089/acm.1995.1.131 9395610

[3] Tuttolomondo T, Licata M, Leto C, Savo V, Bonsangue G, Gargano ML, et al. Ethnobotanical investigation on wild medicinal plants in the Monti Sicani Regional Park (Sicily, Italy). Journal of Ethnopharmacology. 2014;14:153(3):568–86. doi: 10.1016/j.jep.2014.02.032 24632020

[4] Farnsworth NR. The role of ethnopharmacology in drug development. InCiba Foundation Symposium 154‐Bioactive Compounds from Plants: Bioactive Compounds from Plants: Ciba Foundation Symposium 154 2007 Sep 28 (pp. 2–21). Chichester, UK: John Wiley & Sons, Ltd. 10.1002/9780470514009.ch2 2086037

[5] Ahmad M, Zafar M, Shahzadi N, Yaseen G, Murphey TM, Sultana S. Ethnobotanical importance of medicinal plants traded in Herbal markets of Rawalpindi-Pakistan. Journal of Herbal Medicine. 2018;1(11):78–89.

[6] Heinrich M, Lardos A, Leonti M, Weckerle C, Willcox M, Applequist W, et al. Best practice in research: consensus statement on ethnopharmacological field studies–ConSEFS. Journal of ethnopharmacology. 2018;30:211:329–39. 10.1016/j.jep.2017.08.015 28818646

[7] Idolo M, Motti R, Mazzoleni S. Ethnobotanical and phytomedicinal knowledge in a long-history protected area, the Abruzzo, Lazio and Molise National Park (Italian Apennines). Journal of Ethnopharmacology. 2010;3: 127(2):379–95. doi: 10.1016/j.jep.2009.10.027 19874882

[8] Mahmood A, Malik RN, Shinwari ZK, Mahmood AQ. Ethnobotanical survey of plants from Neelum, Azad Jammu and Kashmir, Pakistan. Pak. J. Bot. 2011;1:43(1):105–10.

[9] Ghorbani A. Studies on pharmaceutical ethnobotany in the region of Turkmen Sahra, north of Iran:(Part 1): General results. Journal of ethnopharmacology. 2005;31:102(1):58–68. doi: 10.1016/j.jep.2005.05.035 16024194

[10] Miraldi E, Ferri S, Mostaghimi V. Botanical drugs and preparations in the traditional medicine of West Azerbaijan (Iran). Journal of ethnopharmacology. 2001;1:75(2–3):77–87. doi: 10.1016/s0378-8741(00)00381-0 11297838

[11] Sadeghi Z, Kuhestani K, Abdollahi V, Mahmood A. Ethnopharmacological studies of indigenous medicinal plants of Saravan region, Baluchistan, Iran. Journal of Ethnopharmacology. 2014;11:153(1):111–8. doi: 10.1016/j.jep.2014.01.007 24509152

[12] Mosaddegh M, Naghibi F, Moazzeni H, Pirani A, Esmaeili S. Ethnobotanical survey of herbal remedies traditionally used in Kohghiluyeh va Boyer Ahmad province of Iran. Journal of ethnopharmacology. 2012;7:141(1):80–95. doi: 10.1016/j.jep.2012.02.004 22366675

[13] Dolatkhahi M, Yousefi M, Bagher Nejad J, Dolatkhahi A. Introductory study of the medicinal plant species of Kazeroon, Fars province. Journal of Medicinal Herbs,. 2010;1:1(3):47–56.

[14] Safa O, Soltanipoor MA, Rastegar S, Kazemi M, Dehkordi KN, Ghannadi A. An ethnobotanical survey on hormozgan province, Iran. Avicenna journal of phytomedicine. 2013;3(1):64. 25050260 PMC4075690

[15] Nasab FK, Khosravi AR. Ethnobotanical study of medicinal plants of Sirjan in Kerman Province, Iran. Journal of ethnopharmacology. 2014;28:154(1):190–7. doi: 10.1016/j.jep.2014.04.003 24746480

[16] Sadat-Hosseini M, Farajpour M, Boroomand N, Solaimani-Sardou F. Ethnopharmacological studies of indigenous medicinal plants in the south of Kerman, Iran. Journal of Ethnopharmacology. 2017;6:199:194–204. doi: 10.1016/j.jep.2017.02.006 28167292

[17] Hand D, Mannila H, Smyth P. Principles of Data Mining”. The MIT Press. InA comprehensive, highlytechnical look at the math and science behind extracting useful information from large databases 2001 (Vol. 546).

[18] Heydari F, Rafsanjani MK. A review on lung cancer diagnosis using data mining algorithms. Current Medical Imaging. 2021;1:17(1):16–26. 10.2174/1573405616666200625153017 32586255

[19] Elavarasan D, Vincent DR, Sharma V, Zomaya AY, Srinivasan K. Forecasting yield by integrating agrarian factors and machine learning models: A survey. Computers and electronics in agriculture. 2018;1:155:257–82.

[20] Yoo I, Alafaireet P, Marinov M, Pena-Hernandez K, Gopidi R, Chang JF, et al. Data mining in healthcare and biomedicine: a survey of the literature. Journal of medical systems. 2012;36:2431–48. doi: 10.1007/s10916-011-9710-5 21537851

[21] Dongare AD, Kharde RR, Kachare AD. Introduction to artificial neural network. International Journal of Engineering and Innovative Technology (IJEIT). 2012;2(1):189–94.

[22] Tayi GK, Ballou DP. Examining data quality. Communications of the ACM. 1998; 1:41(2):54–7.

[23] Zhen C, Jiang C. Overview of data mining in the era of big data. International Core Journal of Engineering. 2019;1:5(10):136–9

[24] Liu H, Motoda H, editors. Computational methods of feature selection. CRC press; 2007;29.

[25] Aipperspach R, Rattenbury TL, Woodruff A, Anderson K, Canny JF, Aoki P. Ethno-mining: integrating numbers and words from the ground up. Electrical Engineering and Computer Sciences University of California at Berkeley Tech Report. 2006;6.

[26] Axiotis E, Kontogiannis A, Kalpoutzakis E, Giannakopoulos G. A Personalized Machine-Learning-Enabled Method for Efficient Research in Ethnopharmacology. The Case of the Southern Balkans and the Coastal Zone of Asia Minor. Applied Sciences. 2021; 23:11(13):5826.

[27] Rechinger KH. Flora Iranica, vols. 1–178. Akad Druck-U Verlagsanstalt, Graz. 1963;

[28] Zohary M, Feindbrun-Dothan N. 1966–1986. Flora Palaestina, Vols. 1–4. Jeruselam Academic Pres, Israel.

[29] Townsend CC, Guest E, Al-Ravi A. Flora of Iraq. vols. 1–9. Ministry of Agriculture and Agrarian Reform, Baghdad. 1966.

[30] Davis PH. Flora of Turkey. Flora of Turkey. 1965.

[31] Ghahreman A. Flora of Iran. vols. 1–25. Research Institute of Forests and Rangelands, Tehran (in Persian). 1975.

[32] Raunkiaer C. The life-forms of plants and their bearing on geography. The life forms of plants and statistical plant geography. 1934;2–104.

[33] Trotter RT, Logan MH. Informant consensus: a new approach for identifying potentially effective medicinal plants. InPlants and Indigenous Medicine and Diet 2019;16:91–112. Routledge.

[34] Mostafaeipour A, Sedaghat A, Dehghan-Niri AA, Kalantar V. Wind energy feasibility study for city of Shahrbabak in Iran. Renewable and Sustainable Energy Reviews. 2011; 1:15(6):2545–56.

[35] Rokaya MB, Münzbergová Z, Timsina B. Ethnobotanical study of medicinal plants from the Humla district of western Nepal. Journal of Ethnopharmacology. 2010;9:130(3):485–504. doi: 10.1016/j.jep.2010.05.036 20553834

[36] Stankovic MS, Topuzovic M, Solujic S, Mihailovic V. Antioxidant activity and concentration of phenols and flavonoids in the whole plant and plant parts of Teucrium chamaedrys L. var. glanduliferum Haussk. Journal of Medicinal Plants Research. 2010;18:4(20):2092–8.

[37] Tattini M, Gravano E, Pinelli P, Mulinacci N, Romani A. Flavonoids accumulate in leaves and glandular trichomes of Phillyrea latifolia exposed to excess solar radiation. The New Phytologist. 2000;148(1):69–77. doi: 10.1046/j.1469-8137.2000.00743.x 33863030

[38] Aye MM, Aung HT, Sein MM, Armijos C. A review on the phytochemistry, medicinal properties and pharmacological activities of 15 selected Myanmar medicinal plants. Molecules. 2019;15:24(2):293. doi: 10.3390/molecules24020293 30650546 PMC6359042

[39] Nadembega P, Boussim JI, Nikiema JB, Poli F, Antognoni F. Medicinal plants in Baskoure, Kourittenga province, Burkina Faso: an ethnobotanical study. Journal of ethnopharmacology. 2011;27:133(2):378–95. doi: 10.1016/j.jep.2010.10.010 20950680

[40] JU SK MJ KC, Semotiuk AJ Krishna V. Indigenous knowledge on medicinal plants used by ethnic communities of South India. Ethnobotany Research and Applications. 2019;11:18:1–12.

[41] Hachemi N, Hasnaoui O, Bouazza M, Benmehdi I, Medjati N. The therophytes aromatic and medicinal plants of the southern slopes of the mountains of Tlemcen (western Algeria) between utility and degradation. Research Journal of Pharmaceutical, Biological and Chemical Sciences. 2013;4(1):1194–203.

[42] Heidari Rikan M, Malekmoohamadi L. Medicinal plants in Ghasemloo valley of Uromieh. Iranian Journal of Medicinal and Aromatic Plants Research. 2007;23:23(2):234–50.

[43] Qasim M, Gulzar S, Khan MA. Halophytes as medicinal plants. Urbanisation, land use, land degradation and environment. 2011;330–43.

[44] Heinrich M, Ankli A, Frei B, Weimann C, Sticher O. Medicinal plants in Mexico: Healers’ consensus and cultural importance. Social science & medicine. 1998;1:47(11):1859–71. doi: 10.1016/s0277-9536(98)00181-6 9877354

[45] Uprety Y, Asselin H, Boon EK, Yadav S, Shrestha KK. Indigenous use and bio-efficacy of medicinal plants in the Rasuwa District, Central Nepal. Journal of ethnobiology and ethnomedicine. 2010;6:1–0. 20102631 10.1186/1746-4269-6-3 PMC2823594

[46] Jahantab E, Hatami E, Sayadian M, Salahi Ardakani A. Ethnobotanical study of medicinal plants of Boyer Ahmad and Dena regions in Kohgiluyeh and Boyer Ahmad province, Iran. Adv Herb Med. 2018;4(4):12–22.

[47] Che CT, Wang ZJ, Chow MS, Lam CW. Herb-herb combination for therapeutic enhancement and advancement: theory, practice and future perspectives. Molecules. 2013;3:18(5):5125–41. doi: 10.3390/molecules18055125 23644978 PMC6269890

[48] Farnsworth NR, Akerele O, Bingel AS, Soejarto DD, Guo Z. Medicinal plants in therapy. Bulletin of the world health organization. 1985;63(6):965. 3879679 PMC2536466

[49] Esmaeilzadeh M, Moradi B. Medicinal herbs with side effects during pregnancy-An evidence-based review article. The Iranian Journal of Obstetrics, Gynecology and Infertility. 2017; 22:20:9–25.

[50] Nasri H, Shirzad H. Toxicity and safety of medicinal plants. J HerbMed Plarmacol. 2013;2(2):21–2.

[51] Ahmad L, Semotiuk A, Zafar M, Ahmad M, Sultana S, Liu QR, et al. Ethnopharmacological documentation of medicinal plants used for hypertension among the local communities of DIR Lower, Pakistan. Journal of Ethnopharmacology. 2015;4:175:138–46. doi: 10.1016/j.jep.2015.09.014 26392329

[52] Ishtiaq M, Mahmood A, Maqbool M. Indigenous knowledge of medicinal plants from Sudhanoti district (AJK), Pakistan. Journal of ethnopharmacology. 2015;20:168:201–7. doi: 10.1016/j.jep.2015.01.054 25666425

[53] Dharmadasa RM, Akalanka GC, Muthukumarana PR, Wijesekara RG. Ethnopharmacological survey on medicinal plants used in snakebite treatments in Western and Sabaragamuwa provinces in Sri Lanka. Journal of Ethnopharmacology. 2016;17:179:110–27. doi: 10.1016/j.jep.2015.12.041 26724891

[54] Ribeiro RV, Bieski IG, Balogun SO, de Oliveira Martins DT. Ethnobotanical study of medicinal plants used by Ribeirinhos in the North Araguaia microregion, Mato Grosso, Brazil. Journal of ethnopharmacology. 2017;9:205:69–102. doi: 10.1016/j.jep.2017.04.023 28476677

[55] Li DL, Zheng XL, Duan L, Deng SW, Ye W, Wang AH, et al. Ethnobotanical survey of herbal tea plants from the traditional markets in Chaoshan, China. Journal of ethnopharmacology. 2017;9:205:195–206. doi: 10.1016/j.jep.2017.02.040 28249822

[56] Barkaoui M, Katiri A, Boubaker H, Msanda F. Ethnobotanical survey of medicinal plants used in the traditional treatment of diabetes in Chtouka Ait Baha and Tiznit (Western Anti-Atlas), Morocco. Journal of ethnopharmacology. 2017;23:198:338–50. doi: 10.1016/j.jep.2017.01.023 28109915

[57] Fortini P, Di Marzio P, Guarrera PM, Iorizzi M. Ethnobotanical study on the medicinal plants in the Mainarde Mountains (central-southern Apennine, Italy). Journal of Ethnopharmacology. 2016;26:184:208–18. doi: 10.1016/j.jep.2016.03.010 26969402

[58] Adhikari PP, Talukdar S, & Borah A. Ethnomedicobotanical study of indigenous knowledge on medicinal plants used for the treatment of reproductive problems in Nalbari district, Assam, India. Journal of ethnopharmacology. 2018;210:386–407. doi: 10.1016/j.jep.2017.07.024 28733191

[59] Wu H., Yang S., Huang Z., He J., & Wang X. (2018). Type 2 diabetes mellitus prediction model based on data mining. Informatics in Medicine Unlocked, 10:100–107.

[60] Amancio DR, Comin CH, Casanova D, Travieso G, Bruno OM, Rodrigues FA, et al. A systematic comparison of supervised classifiers. PloS one. 2014;24:9(4):e94137. doi: 10.1371/journal.pone.0094137 24763312 PMC3998948

[61] Sokolova M. Learning from communication data: Language in electronic business negotiations (Doctoral dissertation, University of Ottawa (Canada)).

[62] Dinga R, Penninx BW, Veltman DJ, Schmaal L, Marquand AF. Beyond accuracy: measures for assessing machine learning models, pitfalls and guidelines. BioRxiv. 2019;22:743138.

