# Supplementary material for: Exploring the power of data mining for uncovering traditional medicinal plant knowledge: A case study in Shahrbabak, Iran
Source: PLoS One. 2024 Jun 10;19(6):e0303229. doi: 10.1371/journal.pone.0303229 (PMC11164334; doi:10.1371/journal.pone.0303229)
Supplement: S1 File — (DOCX) [file pone.0303229.s002.docx]

**QUESTIONNAIRE**

Date: ……………………………………… Questionnaire no: …………

Name of the interviewee: ______________________________________________________

**Particulars of area:**

GPS reading: _______________________________________________________________

Name of area: ______________________________________________________________

Name of Sub-location/Sub-area: ________________________________________________

Name of Village (exact place): _ ________________________________________________

**Sociodemographic data:**

| Age: |  |
| --- | --- |
| Gender: |  |
| Educational: |  |
| background: |  |

**Plant information:** Plant number: ____

Persian name: ______________________________________________________________

Vernacular name: __________________________________________________________

Botanical name: ____________________________________________________________

Source:

| Collected from wild | Cultivated at home | both |
| --- | --- | --- |

Part(s) used:

| Underground parts | Leaves | Bark | Fruit | Stem | Whole plant |
| --- | --- | --- | --- | --- | --- |

Other: ___________________________________________________________________

Preparation method:

| Decoction | liniment | both | Is material used dry or fresh? |
| --- | --- | --- | --- |

Mod of application:

| Oral | Topical | both |
| --- | --- | --- |

Symptoms: ________________________________________________________________

__________________________________________________________________________

__________________________________________________________________________

__________________________________________________________________________

Any extra information about the disease and/or its symptoms: __________________________________________________________________________

__________________________________________________________________________

What are Side effects of this plant? _____________________________________________

__________________________________________________________________________

Is this plant used in combination with any other plant(s)? ____________________________

__________________________________________________________________________

Name the plant(s), and describe how this combination is prepared: _____________________

__________________________________________________________________________

__________________________________________________________________________

Any other information: ________________________________________________________

___________________________________________________________________________

___________________________________________________________________________

___________________________________________________________________________

**Researcher:** _______________________ **Signature of interviewee:** ________________
